# MicroRNAs as growth regulators, their function and biomarker status in colorectal cancer

**DOI:** 10.18632/oncotarget.6390

**Published:** 2015-11-25

**Authors:** Lina Cekaite, Peter W. Eide, Guro E. Lind, Rolf I. Skotheim, Ragnhild A. Lothe

**Affiliations:** ^1^ Department of Molecular Oncology, Institute for Cancer Research, Oslo University Hospital, Oslo, Norway; ^2^ K.G.Jebsen Colorectal Cancer Research Centre, Oslo University Hospital, Oslo, Norway

**Keywords:** biomarker, colorectal neoplasm, miRNA, metastasis, target gene

## Abstract

Gene expression is in part regulated by microRNAs (miRNAs). This review summarizes the current knowledge of miRNAs in colorectal cancer (CRC); their role as growth regulators, the mechanisms that regulate the miRNAs themselves and the potential of miRNAs as biomarkers. Although thousands of tissue samples and bodily fluids from CRC patients have been investigated for biomarker potential of miRNAs (>160 papers presented in a comprehensive tables), none single miRNA nor miRNA expression signatures are in clinical use for this disease. More than 500 miRNA-target pairs have been identified in CRC and we discuss how these regulatory nodes interconnect and affect signaling pathways in CRC progression.

## INTRODUCTION

Western societies exhibit a high incidence and mortality of colorectal cancer (CRC), accounting for 10% of the total cancer burden with an individual lifetime risk of ∼6% [1, 2]. CRC patients are stratified according to a clinical staging system (I - IV) at the time of diagnosis. Approximately 20–25% of patients present with metastatic disease, and another 25% will develop metastases in the follow-up period. There is a great need to identify precise biomarkers to facilitate the correct diagnosis, treatment and predict or monitor cancer recurrence.

MiRNAs are a non-protein coding class of small regulatory RNAs (22-nucleotides long) that play an essential role in post-transcriptional regulation of gene expression through binding to the 3′-untranslated region (3′-UTR) of protein-coding mRNAs. A single miRNA may regulate multiple target mRNAs and miRNAs are predicted to regulate approximately 60% of the human genes [3]. Identification and annotation of miRNAs ‘parallels’ technology development, and the introduction of *e.g.* deep sequencing technologies have provided tools for detection and discovery of the regulatory RNA species [4]. The recently released miRNA registry database (miRBASE v21, June 2014) reported a total of 1881 human miRNA genes, counting 2588 unique mature sequences. Independent of the challenges we still are facing regarding miRNA detection methods [5], there are no doubt that these molecules play essential roles in diverse cellular processes [6, 7] (Box 1).

Box 1 **Milestones in miRNA discovery related to cancer [8-17]**

**Figure:**
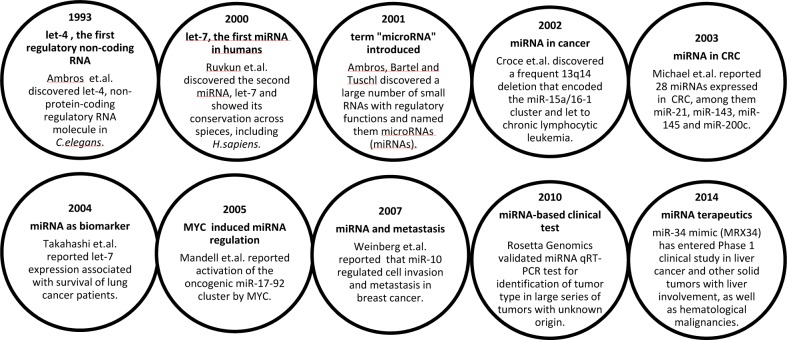

The first evidence associating miRNAs with cancer was demonstrated by the Croce laboratory in 2002, showing that the common 13q14 deletion reduced expression of the miR-15a/16-1 cluster, located within the intron of the *DLEU2* gene, ultimately leading to chronic lymphocytic leukemia [10]. The biological functions of miRNAs are highly dependent on the cellular context, which differ due to diverse compilation of the transcriptome in different tissues and cells. Consequently, and depending on their transcript targets, some miRNAs have increased expression and act as oncogenes in one cancer type, whereas they are downregulated and function as tumor suppressors in another cancer type. Such variability has for instance been seen for let-7, miR-15a/16-1, miR-17-92 cluster, miR-26, miR-29 and miR-125a/b [18, 19]. Therefore, care must be taken when generalizing interpretations of miRNA function across different contexts and tissues.

In this review, we summarize miRNAs that are relevant to CRC, describe research that has led to better understanding of the miRNA function and highlight miRNA involvement in the major signaling pathways.

## OVERVIEW OF MAIN MIRNA RESEARCH AREAS IN CRC

**Figure 1:**
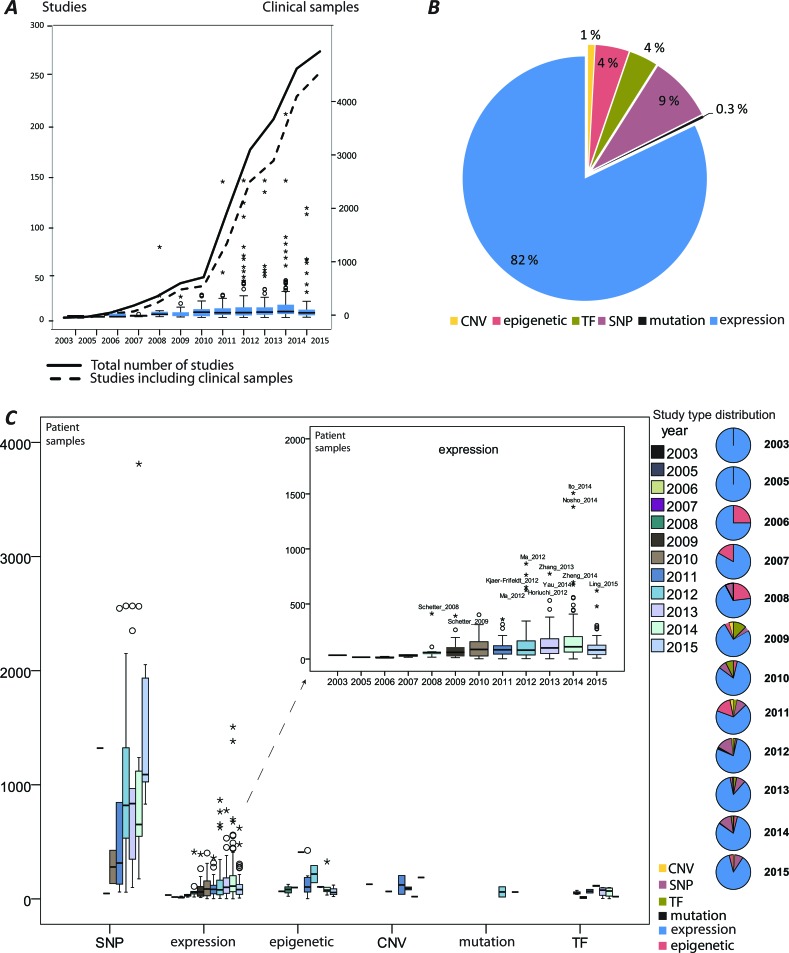
An overview of miRNA studies in colorectal cancer (CRC) **A**. A rapid growth of the published research reports illustrated by the number of studies (with and without clinical samples) per year; the box plots represent numbers of the analyzed patient samples per year; **B**. The proportion of studies reporting aberrant miRNA (expression) and miRNA regulation by copy number variation (CNV), miRNAs regulated by epigenetic modifications, miRNA regulation by transcription factors (TF); single nucleotide polymorphism (SNP) in miRNA coding genes or binding sites of their target genes. **C**. The quantity of patient cohorts segregated by the type of analysis performed.

**Figure 2:**
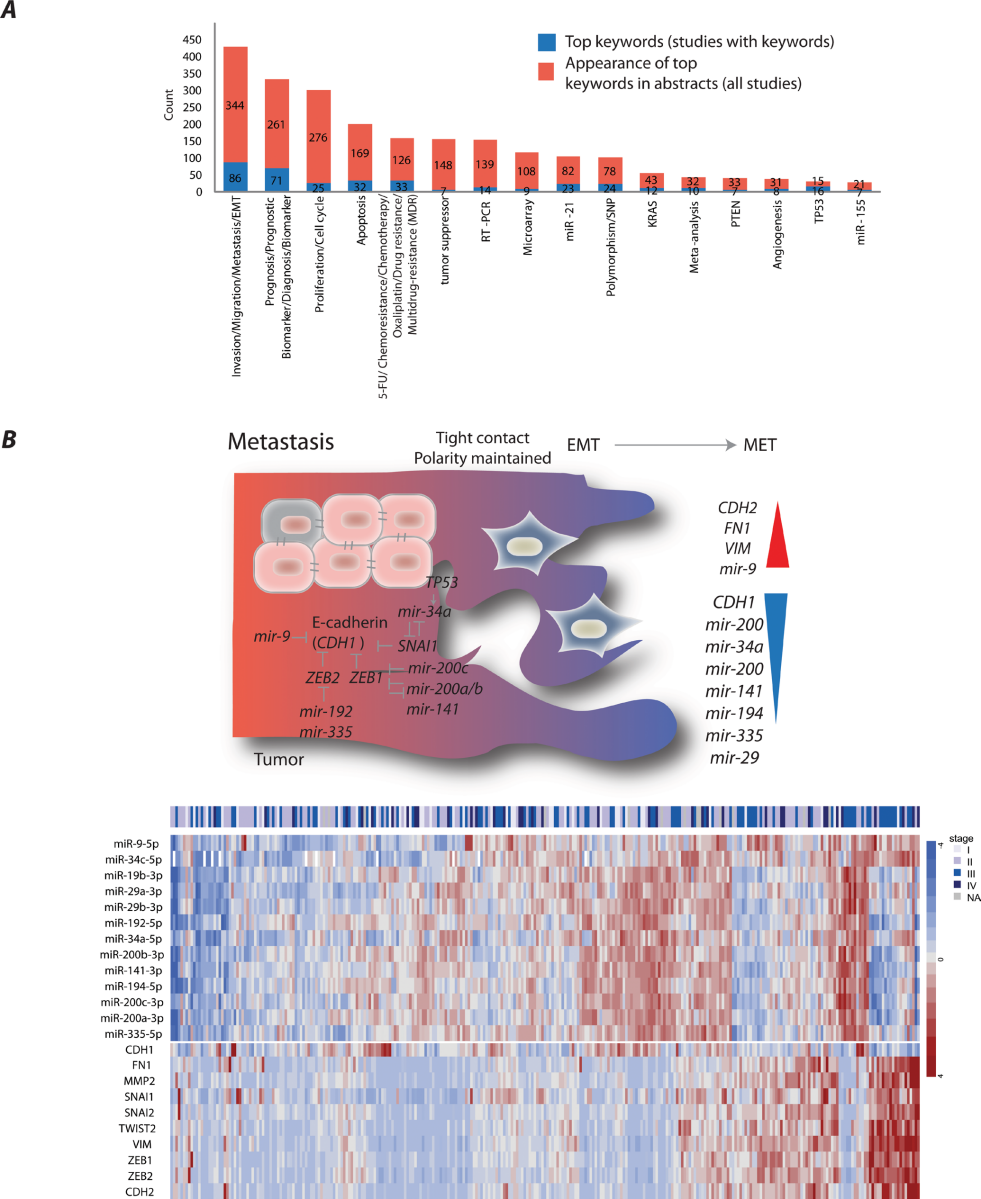
Main miRNA research themes in colorectal cancer (CRC) studies **A**. The trends of miRNA research themes exemplified by article keywords. **B**. An illustration and a heatmap of miRNAs validated to regulate *CDH1*, its regulators and other mesenchymal markers in CRC. The miRNA/mRNA was obtained from CRC patients registered in TCGA. values represent RPM normalized, median-centered values (for further information see Supplementary Material).

MiRNA-induced deregulation in CRC has been well documented and continues to emerge as illustrated by the rapid increase of published studies (information retrieval and handling described in detail in Supplementary Methods) and growing numbers of analyzed clinical samples (Figure 1A). The aberrantly expressed miRNAs and their effects have been primarily addressed (Figure 1B). Approximately 70% of the reports that studied miRNAs in CRC analyzed clinical patient specimens and use of the patient samples increased in recent years. A similar increase in the size of the patient series is not seen (Figure 1C). The mechanisms that deregulate miRNAs, such as single nucleotide polymorphisms (SNPs), epigenetic alterations, mutations, amplifications and loss of genomic regions encoding miRNAs and transcriptional regulation have been addressed to a lower degree (Figure 1B). An overview of the main miRNA research activities in CRC have been generated by recording and ranking the keywords (Figure 2A). The following sections summarize the main research activities and compile the details on the miRNAs' functional role with regards to CRC development and progression.

## MIRNAS DRIVING INVASION, MIGRATION AND METASTASIS

Despite the fact that several miRNAs have been found deregulated early in CRC development, miRNAs have most frequently been described associated with invasion, migration and the progression of disease through epithelial mesenchymal transition (EMT) into metastases (Figure 2A). Cancer cells undergo several molecular changes to generate a mesenchymal cell phenotype necessary for cells to detach and leave the primary tumor. EMT is characterized by loss of cell adhesion, repression of *CDH1*, often caused by transcriptional repression by *SNAI1* [20] and *ZEB1/2* [21], and acquisition of mesenchymal markers, including *CDH2*, *VIM*, and *FN1* [22]. A feed-forward loop consisting of *SNAI1* and miR-34a has been suggested to control the activation of the EMT and mesenchymal-epithelial re-transition programs [23]. The downregulation of miR-34a occurred due to cancer-specific CpG methylation, repression by *IL6R–*mediated *IL-6/STAT3* pathway and/or *TP53* inactivation [23-28]. MiR-34a has also been involved in resistance to 5-FU in part through modulation of glucose metabolism [16]. Furthermore, miR-34a delivery represents a novel therapeutic approach. The first cancer-targeted miRNA drug, MRX34, reinforcing miR-34 expression, has already entered phase I clinical trials in patients with unresectable primary liver cancer and metastatic liver cancers [16]. The trial also includes a separate cohort of patients with hematological malignancies. The preliminary results of this trial have shown manageable safety and a dose-dependent repression of several key oncogenes [29]. MiR-34 has recently been demonstrated to modulate tumor immune evasion pathways through direct downregulation of *PDL1* in lung cancers. MRX34 therapy led to an increase in active tumor-infiltrating immune cells (CD8+) and a decrease in exhausted tumor-infiltrating immune cells (CD8+PD1+) that indicate an active immune response and reduced tumor tolerance [29]. This further supports the potential of miRNAs as therapeutics.

The other *CDH1* repressors, *ZEB1*/*2* were the most prominent targets of the miR-200 family [30-35]. The family includes five members encoded in two clusters: miR-200a/b, miR-429 and miR-200c, miR-141, on chromosomes 1 and 12, respectively. The miR-200b/200a/429 and miR-200c/141 polycistronic transcripts have been shown to be repressed due to CpG methylation [33, 35]. The reduced miR-200 expression was observed at the invasive front of primary CRC that had disrupted basement membrane [34]. In contrast, adenomas and adenocarcinomas with intact basement membranes exhibited uniform miR-200 expression from the tumor core to the tumor-host interface. Interestingly, the metastatic CRCs displayed strong expression of miR-200 and were involved in the recapitulation of the primary tumor phenotype at the metastatic sites [34]. Furthermore, miR-200c increased the sensitivity to chemotherapeutic drugs through repression of *MAPK9* and blocking multidrug resistance [36]. Several other miRNAs, among them, miR-19b, miR-194 and let-7b, had also altered expression when front-specific CRC liver metastases were compared with the tumor center [37].

So far only one study addressed regulation of miR-9 and its direct repression of *CDH1* was confirmed at the protein level in CRC [38]. We previously showed that miR-9 was downregulated at early CRC stages and that its re-expression reduced cell proliferation [39]. However, our and others attempts to establish *CDH1* as miR-9 target in CRC at the mRNA level have failed. This is most likely explained by the fact that miR-9 inhibit *CDH1* translation. Nonetheless, an inverse expression pattern is observed between multiple miRNAs reported to be in control of metastatic progression and selected EMT regulators in TCGA CRC patients [40] (Figure 2B).

The effect of miRNA deregulation on migration and invasion has been frequently measured after perturbations by single synthetic miRNAs in cell line models using wound healing or transfer assays. For example, overexpression of miR-361-5p markedly suppressed proliferation, migration and inhibited tumor growth and lung metastasis *in vivo* [41]. By using a large functional screen, over 20% of miRNAs have been shown to affect migration across diverse cell types, indicating a general involvement of miRNAs in this process [42]. Loss of miR-23b expression in colon cancer resulted in increased activity of prometastatic genes, including *FZD7* and *MAP3K1*, and facilitated tumor growth, invasion and angiogenesis *in vivo* [42]. By employing “dropout” screens of a miRNA library in a mouse model of liver metastasis, Okamoto and colleagues have shown that upregulation of miR-493 during carcinogenesis prevented liver metastasis via the induction of cell death of metastasized cells [43]. To colonize the liver, CRC metastatic lesions must overcome hypoxia and energy starvation. Tumor cells have been demonstrated to release a metabolic enzyme *CKB*, upregulated through loss of miR-551a and miR-483-5p, into the extracellular matrix and catalyzing liver metabolites into ATP-rich products, which further were taken up by the cell to fuel metastatic survival and subsequent liver colonization [41].

The crucial miRNA function in metastasis is also supported by a meta-analysis employing TCGA data across multiple cancer types, including CRC, that reported metastasis as the most prominent process under miRNA regulation [44].

## MIRNAS WITH BIOMARKER POTENTIAL

Due to high tissue specificity, stability and altered expression in neoplastic development, miRNAs have been suggested as diagnostic, prognostic and predictive biomarkers. The Cancer Origin Test, based on the expression of 64 miRNAs, is the first promising assay available for determining the tumor origin with 90% sensitivity and 99% specificity for metastatic cancer of unknown or uncertain primary site [15]. MiRNA-based tests for improved subclassification of lung, kidney cancers and mesothelioma subtypes are also on the market [45]. However, despite the long list of miRNAs suggested as biomarkers in CRC, none have made it to the clinic yet. The individual studies that have reported miRNAs as circulating biomarkers in blood serum or plasma and feces as well as prognostic and/or predictive biomarkers in tumor tissues in CRC are summarized in Tables 1 and 2. Approximately 40% of the reported CRC miRNA biomarkers were suggested by two or more studies, among them, miR-21, miR-29a, miR-92a, miR-200c, miR-215 and miR-375.

**Table 1:** Tissue miRNAs as potential biomarkers for Colorectal Cancer (CRC)

Application	miRNA biomarker	Type of analysis*	Total nr of samples#	Ref.
	MIRNA BIOMARKERS IN TISSUE BIOPSIES		specimens ^#^	
Anti-angiogenetic therapy	miR-126	↑	109	[172]
Anti-EGFR therapy	let-7a	↑	59	[173]
	let-7c, miR-99a, miR-125b	signature	183	[174]
	miR-7	↓	105	[58]
	miR-31-5p	↑	102	[175]
	miR-125b, miR-137	↑	66	[176]
Chemotherapy	let-7g, miR-132, miR-224, miR-320a	signature	128	[177]
	miR-16, miR-590-5p, miR-153, miR-519c-3p, miR-561	signature	32	[178]
	miR-17-5p	↑	390	[179]
	miR-21	↑	84	[180]
	miR-21	↑	301	[181]
	miR-21, miR-143, miR-145	expression, diverse	120	[116]
	miR-107, miR-99a-3a	↑	78	[182]
	miR-1183, miR-483-5p, miR-622, miR-125a-3p, miR-1224-5p, miR-188-5p, miR-1471, miR-671-5p, miR-1909*, miR-630, miR-765, miR-622, miR-630	signature	37	[183]
	miR-126	expression, not significant	158	[184]
	miR-143	↓	175	[185]
	miR-148a	↓, methylation	293	[186]
	miR-148b	↓	192	[187]
	miR-150	↓	625	[54]
	miR-181d, miR-139-3p, miR-892b, miR-338-5p, miR-150, miR-154,miR-454	signature	70	[188]
	miR-200a, miR-200c, miR-141, miR-429	↓	127	[189]
	miR-200c	↓	103	[190]
	miR-214	↓	242	[191]
	miR-215	↑	48	[192]
	miR-215, miR-190b, miR-29b-2*, miR-196b, let-7e, miR-450a, miR-450b-5p, miR--99a*	signature	20	[193]
	miR-320	↓	100	[194]
	miR-451	↓	35	[195]
	miR-622	↑	17	[55]
	miR-625-3p, miR-181b, miR-27b	↑	283	[196]
Diagnosis	miR-21	↑	360	[197]
	miR-21	↑	73	[198]
	miR-31	↑	1382	[46]
	miR-34b	↓, methylation	113	[199]
	miR-155	↑	80	[200]
	miR-194	↓	446	[201]
	miR-375, miR-424, miR-92a	signature	282	[64]
	miR-378*, miR-145	↓	64	[202]
Prognosis/Survival /Metastasis	miR-21	↑	554	[203]
	miR-21	↑	412	[204]
	miR-21	↑	392	[205]
	miR-21	↑	7 studies	[206]
	miR-21	↑	14 studies	[207]
	miR-21, miR-155	↑	312	[208]
	miR-21-5p, miR-20a-5p, miR-103a-3p, miR-106b-5p, miR-143-5p, miR-215	signature	775	[209]
	let-7a	↓	40	[210]
	let-7c	↓	254	[211]
	miR-1, miR-129, miR-215, miR-497, miR-135b, miR-493	signature	49	[212]
	miR-101	↓	36	[213]
	miR-106a, miR-143, miR-125b	expression, diverse	48	[214]
	miR-126	↓	560	[215]
	miR-130b	↑	160	[216]
	miR-139-3p	↓	126	[217]
	miR-139-5p, miR-31, miR-17-92, miR-143, miR-10b	signature	142	[218]
	miR-143	↓	154	[219]
	miR-148a, miR-152	↓	202	[220]
	miR-15, miR-16	↓	126	[221]
	miR-15b, miR-135b	↓	189	[222]
	miR-17-92, miR-17	↑	48	[223]
	miR-182	↑	296	[52]
	miR-182	↑	176	[75]
	miR-183	↑	188	[224]
	miR-195	↓	170	[225]
	miR-195, miR-1280, miR-140-3p, miR-1246	signature	19	[226]
	miR-196a, miR-196b	↑	252	[227]
	miR-200a, miR-17, miR-106a, miR-375, miR-29a, miR-18a, miR-200b	signature	267	[228]
	miR-215	↑	116	[229]
	miR-224	↑	230	[230]
	miR-338-3p	↓	80	[231]
	miR-340	↓	155	[232]
	miR-34-5p	↓	278	[28]
	miR-375	↓	190	[233]
	miR-429	↑	214	[234]
	miR-638	↓	312	[70]
	miR-21	↑	20	[235]
	miR-21, miR-103, miR-93, miR-566	signature	187	[236]
	miR-25-3p, miR-339-5p	↓	93	[237]
	miR-27a	↓	82	[238]
	miR-122	↑	12	[239]
	miR-133a	↓	338	[240]
	miR-133b	↓	62	[241]
	miR-133b	↓	100	[242]
	miR-185	↓	40	[243]
	miR-185, miR133b	expression, diverse	50	[244]
	miR-193a-5p	↓	360	[245]
	miR-199a-5p	↓	20	[246]
	miR-200b, miR-200c	↓	13	[247]
	miR-210	↑	193	[248]
	miR-210, miR-133b	expression, diverse	54	[249]
	miR-224	↓	158	[250]
	miR-224, miR-221*	↓	60	[251]
	miR-625	↓	192	[252]
	miR-21	↑	277	[253]
	miR-29a	↓	110	[254]
	miR-215	↓	125	[255]

* ↑, upregulated expression in CRC vs. normal control; ↓, downregulated expression in CRC vs. normal control; methylation, cancer specific DNA methylation; signature, multiple miRNAs considered collectively; expression, diverse, multiple miRNAs considered separately, #total number of samples included in the study, CRCs, adenomas and normal control samples collectively; specimens #1 – tissue samples from normal colonic mucosa, adenomas and cancer biopsies.

**Table 2:** Circulatory miRNAs as potential biomarkers for Colorectal Cancer (CRC)

Application	miRNA biomarker	Type of analysis*	Total nr of samples#	Ref.
	MIRNA BIOMARKERS IN SERUM/PLASMA		specimens #1	
Anti-angiogenetic therapy	miR-126	↑, SNP	178	[256]
Anti-EGFR therapy	miR-345	↑	138	[257]
Chemotherapy	miR-19a	↑	88	[258]
	miR-20a, miR-130, miR-145, miR-216, miR-372	signature	253	[118]
	miR-27b, miR-148a, miR-326, miR-106a, miR-484, miR-130b	signature	328	[259]
	miR-126	↑	178	[260]
	miR-143	↓	51(175)	[185]
	miR-155, miR-200c, miR-210	↑	35	[261]
Diagnosis	miR-17-3p, miR-29a, miR-92a, miR-135b	expression, not significant	130	[262]
	miR-18a, miR-19a, miR-19b, miR-15b, miR-29a, miR-335	signature	196	[263]
	miR-19a-3p, miR-223-3p, miR-92a-3p, miR-422a	signature	697	[264]
	miR-21	↑	5 studies	[62]
	miR-21	↑	366(532)	[265]
	miR-21	↑	100(192)	[59]
	miR-21	↑	75(189)	[60]
	miR-21	↑	80	[266]
	miR-21	↑	71	[267]
	miR-21, let-7g, miR-31, miR-92a, miR-181b, miR-203	signature	202	[268]
	miR-21, miR-378	↑	135	[269]
	miR-21, miR-92a	↑	98	[270]
	miR-29a, miR-92	↑	216	[271]
	miR-34a	↓	108	[272]
	miR-92	↑	255(265)	[273]
	miR-145	↓	50(170)	[274]
	miR-145	↓	60	[275]
	miR-193a-3p, miR-23a, miR-338-5p	signature	162(222)	[276]
	miR-375, miR-206, miR-150, miR-125b, miR-126*	↓	140(228)	[277]
	miR-409-3p, miR-7, miR-93	signature	241	[278]
	miR-423-5p, miR-210, miR-720, miR-320a, miR-378, miR-106a, miR-143, miR-103, miR-199a-3p, miR-382, miR-151-5p	signature	90	[279]
	miR-423-5p, miR-484	, diverse	103	[280]
	miR-532-3p, miR-331, miR-195, miR-17, miR-142-3p, miR-15b, miR-532, miR-652, miR-431, miR-15b, miR-139-3p	signature	128	[281]
	miR-574-5p	↑	14(146)	[281]
	miR-601, miR-760	↓	191	[282]
Prognosis/Survival/ Metastasis	miR-7, miR-17-3p, miR-20a, miR-21, miR-92a, miR-96, miR-183, miR196a and miR-214, miR-124, miR-127-5p, miR-138, miR-143, miR-146a, miR-222	expression, diverse	20(40)	[283]
	miR-21	↑	102	[284]
	miR-124-5p, miR-26a	↓	71(142)	[285]
	miR-141	↑	278	[286]
	miR-155	↑	206	[287]
	miR-182	↑	51(161)	[66]
	miR-200c	↑	230(446)	[288]
	miR-218	↓	90(331)	[289]
	miR-221	↑	140	[61]
	miR-23a	↑	102(162)	[290]
	miR-29a, miR-92a	↑, no associations for miR-92a	74(114)	[291]
	miR-92a	↑	56(94)	[292]
	let-7i, miR-10b, miR-221, miR-320a, miR-885-5p	signature	169(478)	[293]
	miR-126, Let-7a, miR-141, miR-21	signature	224	[294]
	miR-15a, mir-103, miR-148a, miR-320a, miR-451, miR-596	signature	40	[295]
	miR-18a, miR-29a	↑	56(80)	[296]
	miR-29c	↑	84(191)	[297]
	miR-148a	↓	85(195)	[298]
	miR-183	↑	179(195)	[65]
	MIRNA BIOMARKERS IN STOOL		specimens #2	
Diagnosis	miR-7, miR-17, miR-20a, miR-21, miR-92a, miR-96, miR-106a, miR-134, miR-183, miR-196a, miR-199a-3p, miR-214, miR-9, miR-29b, miR-127-5p, miR-138, miR-143, miR-146a, miR-222, miR-938	signature	60(75)	[299]
	miR-21, miR-106a	↑	37(42)	[300]
	miR-34b, miR-34c	methylation	122(286)	[301]
	miR-34b, miR-34c, miR-148a	methylation	67(189)	[302]
	miR-106a	↑	224	[303]
	miR-135b	↑	424(490)	[304]
	miR-143, miR-145	↓	51	[305]
	miR-144*	↑	75(105)	[306]
	miR-221, miR-18a	↑	595(675)	[307]
	miR-451, miR-223	↑	45(61)	[308]
	miR-J1-5p	↓	56(68)	[309]
	miR-21, miR-92a	↑	239(326)	[310]
	miR-135a, miR-135b, miR-18a, miR-19a	↑	340(402)	[311]
	miR-20a-5p, miR-21-3p, miR-141	↑	40(80)	[312]

* ↑, upregulated expression in CRC vs. normal control; ↓, downregulated expression in CRC vs. normal control; methylation, cancer specific DNA methylation; signature, multiple miRNAs considered collectively; expression, diverse, multiple miRNAs considered separately, #total number of samples included in the study, CRCs, adenomas and normal control samples collectively; specimens #1 – serum or plasma samples from healthy controls, individuals with adenomas and cancer patients, total number of samples, including tissue samples are given in parenthesis; specimens #2 - stool samples from healthy controls, individuals with adenomas and cancer patients, total number of samples, including tissue samples are given in parenthesis.

### miRNA tissue biomarkers

Aberrantly expressed miRNAs have been found in adenomas and carcinomas of the large bowel as compared to normal colonic mucosa, and several of these miRNAs have been suggested as biomarkers. A high miR-31 expression was observed in serrated lesions and suggested as diagnostic biomarker and therapeutic target for *BRAF* mutated CRCs [46]. A signature of miR-92a, miR-375 and miR-424 has been shown to discriminate invasive carcinoma from adenoma with high-grade neoplasm in biopsies from colonoscopy, representing promising clinical utility in early diagnosis [47]. A predictive value of miR-29a, where high levels of this were associated with longer disease-free survival, has been shown in patients with stage II CRC [48]. With few exceptions, stage II CRC patients are treated with surgery alone. If considered to have a high risk of relapse based on clinicopathological parameters they may be offered adjuvant chemotherapy [49]. A 6 miRNA classifier, consisting of miR-21-5p, miR-20a-5p, miR-103a-3p, miR-106b-5p, miR-143-5p, and miR-215 has been shown to distinguish between stage II colon cancer patients with high and low risk of disease progression, with 5-year disease-free survival of 60% and 89%, respectively [50]. The miRNA panel outperformed clinicopathological risk factors and DNA mismatch repair status when it came to predicting the prognosis, and patients in the high-risk group were found to have a favorable response to adjuvant chemotherapy [50]. Furthermore, stromal expression of miR-21 alone was found as an independent predictor of early tumor relapse in a large colon cancer series, indicating that these patients could be considered for more intensive treatment [45].

As mentioned above, miR-34a and miR-200 family members have a functional role in CRC metastases. Supporting this notion, miR-34a has also been suggested as an independent predictor of recurrence among stage II/III patients [28], while expression levels of miR-200 family members identified those CRC patients, including stage II, who are most likely to benefit from adjuvant chemotherapy [51]. High expression of miR-182, identified as anti-apoptotic miRNA [39], has been associated with lymph node metastases and poor survival, and suggested to have clinical potential not only as a promising predictor of aggressive phenotype but also as an independent prognostic predictor to identify patients with particularly low rate of survival [52]. Interestingly, anti-miR-182 therapy has been demonstrated as a promising therapeutic strategy for metastatic melanoma [53].

Drug resistance is the major obstacle of effective cancer therapy, and a number of miRNAs have been shown to induce chemoresistance and to be associated with poor prognosis. Patients whose tumors had low miR-150 expression exhibited shorter survival and a worse response to adjuvant chemotherapy [54]. Overexpression of miR-622 was induced by radiotherapy in rectal cancers, causing poor response to therapy [55]. A signature of miR-99a, let-7c and miR-125b distinguished patients with *KRAS* wild-type metastatic CRC that responded to anti-EGFR therapy [56]. Reduced expression of miR-181a was associated with poor clinical outcome in patients treated with EGFR inhibitor [57]. Whereas, reduced levels of miR-7, a direct regulator of *EGFR*, was suggested as a prognostic biomarker and a candidate for targeted therapy in patients with CRC whose tumors were resistant to targeted anti-EGFR therapy [58]. Despite the fact that miRNA tissue biomarkers offer great promise, the miRNA biomarkers' clinical significance is not conclusive and independent validation studies are needed.

### Circulating miRNA biomarkers

The diagnostic and prognostic potential of circulating miRNAs have in recent years also been evaluated in blood derivatives (plasma or serum) and feces. Considering that a single cell may express hundreds of copies of a single miRNA, cancers may at least in principle be detected earlier with a miRNA- compared to a DNA-based test. In addition, in circulation, tumor-derived miRNAs appear to be protected from degradation by endogenous ribonucleases. Thus miRNAs are promising candidates for minimally invasive biomarkers.

For example, high levels of serum miR-21 distinguished patients with adenomas and CRCs from healthy control subjects [59-61], however, a meta-analysis has shown moderate sensitivity of 77% and 85% specificity [62]. Interestingly, a higher concentration of miR-21 was found in the blood drawn near to the site of the primary tumor compared to peripheral blood, indicating that the primary tumor releases a high number of miR-21, which is diluted in the circulatory system [60]. Plasma levels of miR-92 distinguished patients with CRC from gastric cancer, inflammatory bowel disease and healthy control subjects [63]. A high diagnostic accuracy has been shown by a panel of miR-409-3p, miR-7, and miR-93 in discriminating CRC from controls with 91% sensitivity and 88% specificity [64]. Although plasma miR-183 alone displayed moderate sensitivity (74%) and specificity (86%) in detecting CRCs, it outperformed carcinoembryonic antigen (CEA) and carbohydrate antigen 19-9 (CA19-9) [65]. Furthermore, the postoperative plasma miR-183 levels were significantly reduced compared with the preoperative levels [65]. Similarly, miR-182 was detected in plasma from metastatic CRC patients and the expression levels were reduced in post-operative samples after radical hepatic metastasectomy compared to preoperative samples [66]. Plasma miR-27b, miR-148a, and miR-326 levels predicted response to 5-FU and oxaliplatin-based chemotherapy in metastatic CRC patients [67].

MiRNAs have been analyzed in fecal samples from CRC patients and from healthy controls. Fecal miR-106a detection has been shown to improve sensitivity of immunological fecal occult blood test (iFOBT) with a combined sensitivity of 71% and a specificity of 96% [68]. The expression of miR-17-92 cluster and miR-135 were significantly higher in CRC patients than in healthy controls, and discriminated CRC with an overall sensitivity and specificity of 74% and 79% [69].

Despite the high biomarker potential of individual miRNAs, it is likely that panels of miRNAs more accurately can identify patients at risk. Furthermore, combined biomarker panels, comprising miRNA, mRNA and/or DNA methylation markers may also outperform one-level biomarkers. Nevertheless, evaluation of biomarker panels using large, independent patient cohorts must be performed before miRNA biomarkers can implemented in the clinic.

## MIRNAS REGULATING PROLIFERATION AND APOPTOSIS

The effect of miRNAs on cellular proliferation could be subdivided into miRNAs targeting 1) mitogen receptor tyrosine kinases, or G-protein signal transducers such as *KRAS*, and the intermediary signal transducing molecules that convey mitogenic information to its intracellular targets, 2) cell cycle regulators, such as late-G1 checkpoint regulators *RB* and *CDKs,* and 3) reciprocal miRNA interactions with *MYC* and *TP53*. The comparisons of the proliferative potential of two cell populations, frequently addressed in many studies, may reflect not only efficiency of cell cycle, but also cell differentiation and senescence, survival or apoptosis. In addition, cell proliferation and metabolism are tightly linked cellular processes, since cells need to increase their biomass and replicate their genome prior to proliferation. The effect of miRNAs on apoptosis is mediated by their regulation of pro-apoptotic and anti-apoptotic mRNAs or their positive regulators. Augmented cell proliferation together with suppressed apoptosis constitutes the minimal common platform upon which all neoplastic development occurs. MiRNAs with pro-proliferative and/or anti-apoptotic activities would likely promote oncogenesis and thus may be overexpressed in cancer cells. Likewise, miRNAs with anti-proliferative and/or pro-apoptotic activities are likely to function as tumor suppressor genes and thus may be downregulated in cancer cells.

The effect of miRNA deregulation on cell proliferation and apoptosis has frequently been measured after perturbations of single miRNAs in cell line models. The ectopic expression of miR-638 inhibited CRC cell proliferation by targeting *TSPAN1* and arresting the cell cycle in G1 phase [70]. Overexpression of miR-29b induced apoptosis and arrested the cell cycle in the G1/S transition by repressing *MCL1* and *CDK6* [71]. High throughput approaches analyzing the effect of multiple miRNAs simultaneously, have also been performed [39, 43, 72]. The perturbations of entire population of miRNAs by employing a comprehensive miRNA library screen, have shown that approximately 15% and 30% of miRNAs had an effect on proliferation and apoptosis, respectively [39]. However, only some of these are relevant to CRC development and progression. By integrating miRNA expression from CRC patients and applying in-depth functional validation, the strongest candidates have been identified, including miR-9, miR-31, miR-182, miR-375 and miR-491 [39, 72, 73]. MiR-491 induced apoptosis via silencing of *BCL2L1* that is commonly overexpressed in CRC, and treatment with miR-491 suppressed *in vivo* tumor growth [72]. Interestingly, the genomic micro-deletions at chromosome 9 (cytoband 9p21.3) and chromosome 20 (cytoband 20q13.33) that include the mir-491/*KIAA1797* and *AK309218*/mir-646 genes, respectively, have been associated with familial and early-onset CRC [74]. Enhanced expression of miR-182 in CRC has been reported in multiple studies [39, 52, 75, 76] and associated with lymph node metastasis and poor prognosis [52, 75]. MiR-182 exhibited anti-apoptotic effect by targeting *ENTPD5 [77], FBXW7, SATB2 [78]* and *THBS1 [79],* and stably blocking its expression in animal models has been shown to inhibit tumor formation [80].

## MIRNA ABERRATIONS IN THE MOLECULAR PHENOTYPES OF CRC

CRCs are primarily subdivided into three molecular phenotypes. The chromosomal instability phenotype (CIN) accounts for about 85 % of all sporadic CRCs and is characterized by large-scale genomic rearrangements [81]. The microsatellite instability (MSI) phenotype accounts for about 15 % of all CRCs and is associated with a defect DNA mismatch-repair system (MMR), and an accordingly hypermutated genome [82, 83]. Cancers with the CpG island methylator phenotype (CIMP) display promoter hypermethylation and subsequent inactivation of multiple genes. CIMP cancers overlap with both the CIN and MSI types, but are mainly associated with MSI [84]. Recently, transcriptome instability (TIN) was introduced as a fourth molecular phenotype, characterized by excess aberrant pre-mRNA splicing [85].

The impact of abnormalities of the miRNA-ome is still limited for these individual molecular phenotypes. Most studies have focused on comparison of the most common phenotypes CIN to MSI where differential expression of miRNAs has been reported. Further, in MSI cancers, expression levels of certain miRNAs have been associated with resistance to chemotherapy [86-93]. Furthermore, miR-155 and miR-21, which are frequently overexpressed in CRC, have been demonstrated to down-regulate the core MMR proteins MSH2, MSH6, and MLH1, inducing the MSI mutator phenotype [88, 92, 94]. MiR-155 has been found overexpressed in MSI tumors with unknown cause of MMR inactivation and therefore suggested as a potential mechanism of MSI induced cancer pathogenesis [88]. The MSI phenotype is characterized by a dramatic increase of insertions and deletions at repetitive sequences. Even though mutated DNA repeats in miRNA hairpin sequences are relatively rare, cancers with MSI phenotype also have an elevated rate of single nucleotide mutations. Thus, miRNA genes are as well potential mutation targets MSI CRCs. A single miRNA, miR-1303, displayed frequent mutations due to MSI and was expressed in colonic tissues [95]. Functional studies are required to conclude whether this miRNA might have a role in MSI tumorigenesis.

MiRNAs are frequently located at fragile sites of the genome, which are usually either amplified or deleted in human cancers [96]. The aberrant miRNA expression in CRC could be due to these genomic rearrangements associated with CIN phenotype. DNA copy number gain on chromosome band 13q31 has been shown to increase the expression of the oncogenic miR-17-92 cluster [97]. Reduced expression of miR-497 and miR-195 in CRC was associated with DNA copy number loss of a segment of chromosome band 17p13.1 [98]. However, to which extent these miRNAs are oncogenic drivers for cancers with the recurring rearrangements is not proven.

One of the most common causes of the loss of tumor suppressor miRNAs in CRC is the silencing of their primary transcripts by cancer-specific DNA methylation in associated CpG islands [99-102]. However, there is no evidence whether more miRNA genes are affected in CIMP positive tumors displaying higher frequency and extent of DNA methylation as compared to other subtypes. So far, only two miRNAs, miR-31 [103] and miR-146a [104], have been associated with the CIMP positive CRCs.

## MIRNA REGULATION OF TARGET GENES

**Figure 3:**
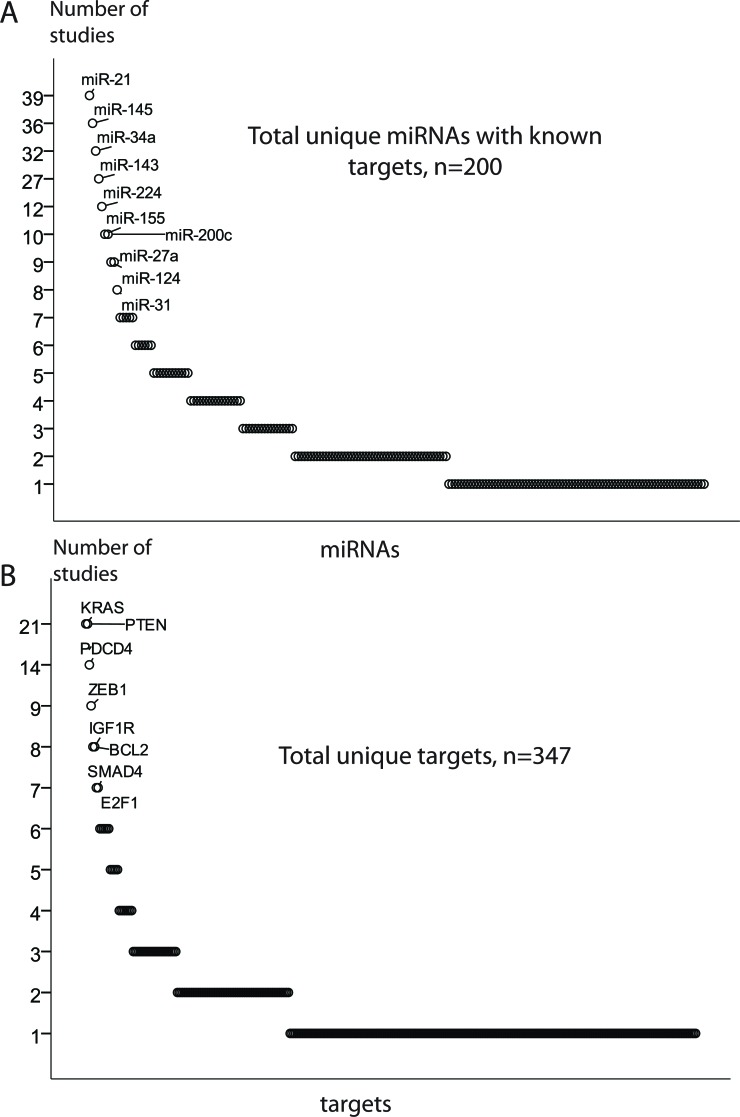

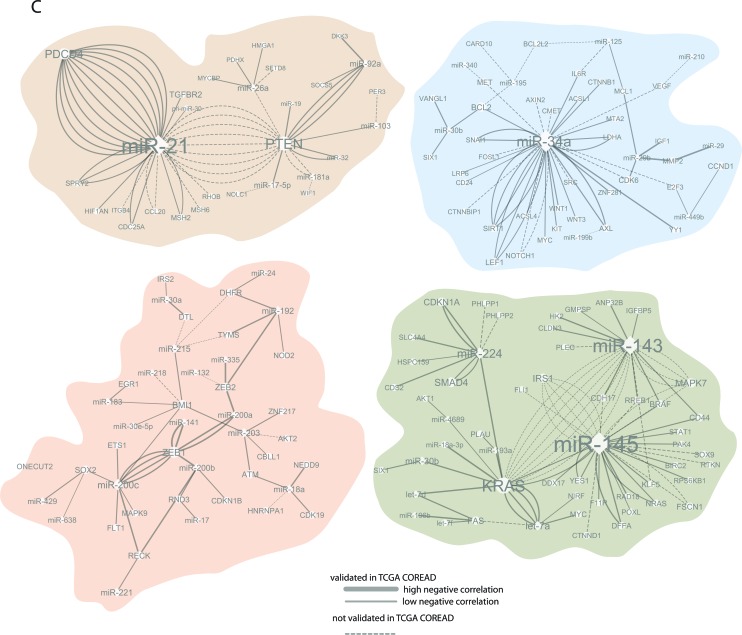
MiRNA regulation of target genes **A**. and **B**. The most frequently validated miRNA and target genes. **C**. The enlarged leading miRNA-targets nodes generated using the validated miRNA-target pairs. The network edges are coded by degree of expressional miRNA-mRNA associations obtained in TCGA CRC patients. MiRNA nodes are indicated by rectangles, target nodes as ovals. Size of nodes reflects the number of connections, with bigger nodes representing more densely connected regions. The node of miR-143/miR-145-KRAS should be taken with precaution in light of the recent results by Chivukala *et. al*.[105].

Approximately 450 unique miRNAs have been associated with CRC, of which twenty account for one third of all miRNA quotations. MiRNAs miR-21, miR-143 and miR-145 were the most frequently reported, followed by miR-31, miR-34a, miR-200c, miR-20a and miR-92a. Recent experimental analyses have validated a total of 530 miRNA-mRNA pairs in CRC, 200 unique miRNAs and 347 unique targets (Figure 3A-3B).

### Modeling cross-talk in miRNA and their target networks based on validated interactions

Most of the studies reporting miRNAs and their targets in CRC validated only a single miRNA-mRNA pair, despite that 1) miRNAs may have target sites in multiple transcripts and 2) several miRNAs may play together in controlling a single mRNA transcript. To explore the underlying regulatory circuits of miRNAs and their targets, we extracted all experimentally validated interactions and built a miRNA-target network by combining all miRNA-mRNA pairs reported in the reviewed studies. The four nodes of miR-21-*PTEN*/*PDCD4*, miR-143/miR-145-*KRAS*-let-7a, miR-34a-*SIRT1/LEF1* and miR-200c-*ZEB1* were prominent in the miRNA-target interaction network. In addition, a small number of miRNA-mRNA nodes included two to four partners, and the bulk of miRNAs that so far have reported with only one validated target. The miRNA-target interaction network is available in Supplementary Figure S1, and selected enlarged nodes are highlighted in Figure 3C. The node of miR-143/miR-145-KRAS should be interpreted with precaution in light of the recent results by Chivukala *et. al.* [105] (further discussed on page 13).

Although the network analysis illustrates the complexity of miRNA-induced regulation, it is strongly biased towards well-studied protein coding genes and miRNAs. Since the miRNA functions are dependent on available cellular transcripts, the cell composition of a tumor may further influence this interaction map. Moreover, some of the miRNAs targets may be repressed more strongly than others. Ideally, one should therefore predict not only miRNA-target pairs, but also the expected degree of translational suppression, taking into account seed complementarity and mRNA secondary structure [106]. Recently, the distribution of the recurrent miRNA-target interactions have been analyzed employing TCGA data across multiple cancer types, including CRC [44, 107]. We have integrated miRNA-mRNA associations obtained in the TCGA CRC patients [44] with the network of validated miRNA-target pairs. More than 60% of validated miRNA-target interactions collected across all reviewed studies had, as expected, negative expression associations in the TCGA CRC patients (Figure 3C). This also suggests that a part of these experimentally validated interactions in CRCs and/or CRC cell lines had no association or even displayed positive associations in the TCGA CRC patients. This might be explained by some targets that reciprocally control the level and function of miRNAs. Furthermore, we cannot exclude the patient cohort specificity, or that some miRNA-mRNA interactions validated in CRC cell lines only, are not relevant to CRC patients.

## MIRNAS REGULATE KEY COLORECTAL CANCER SIGNALING PATHWAYS

**Figure 4:**
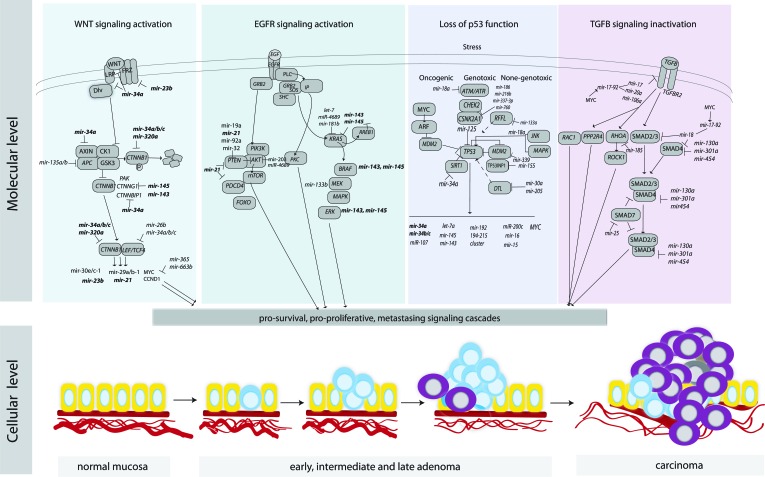
An overview of key signaling pathways in CRC and the regulation of their components by miRNAs MiRNAs that are involved in multiple pathways and contribute to the cross-talk among the pathways are marked in bold.

CRC tumorigenesis is driven by molecular alterations resulting in activation of pro-survival signaling pathways such as the canonical WNT signaling pathway, the EGFR pathway, the TP53 network, and the transforming growth factor beta (TGF-β) (Figure 4).

### Activation of canonical WNT signaling

WNT signals are pivotal for the regulation of stem cell activity to the crypt bottom in the intestines and ultimately for the renewal of the epithelial cells [108]. Inappropriate activation of WNT signaling promotes cell survival, inhibits cell death and differentiation and triggers the development of upper- and lower- gastrointestinal polyps and carcinoma [109]. The APC tumor suppressor is a negative regulator of free CTNNB1, a central player of WNT signaling, and up to 80% of CRCs have mutations in the *APC* gene resulting in a truncated protein [110]. MiRNAs may also contribute to reduced *APC* levels. The elevated expression of miR-135a/b, negative regulator of *APC*, has been observed in adenocarcinomas as well as premalignant colorectal adenomas and correlated with concomitant reduced levels of *APC* leading to WNT pathway activation [111]. Interestingly, miR-135a/b suppressed *APC* expression, even when *APC* mutations were present, suggesting that miR-135a/b may function as complementary players in WNT pathway addiction. Furthermore, overexpression of miR-135b, activated by the WNT pathway, has been observed in sporadic as well as inflammatory bowel disease-associated CRCs and correlated with tumor stage and poor clinical outcome [112].

A critical point of WNT signaling is nuclear translocation of CTNNB1 that binds to the LEF/TCF transcription complex and activates transcription of multiple target genes, including *CCND1* and *MYC* [113]. MiR-26b and miR-34a/b/c directly targeted *LEF1* 3′UTR and inhibited its expression [114], while miR-145 directly targeted *CTNNG1*, contributing to the aberrant translocation of CTNNB1 through impaired nuclear shuttling with PAK4 [115]. A reduced miR-143/145 expression in CRC [11] was reported in the first study associating miRNA and CRC, and roughly 40 other studies have reproduced these results. Nevertheless, the tumor suppressor role of miR-143/145 in CRC has been challenged by the recent results of Chivukula and colleagues, who reported that miR-143/145 were expressed exclusively within the mesenchymal compartment of the normal intestine and that the repeatedly observed repression of miR-143/145 in CRC was rather the result of depletion of mesenchymal cells in tumors relative to normal mucosal biopsies [105]. Nonetheless, loss of miR-143/145 expression has been shown to predict poor prognosis and serve as CRC biomarkers [116-118]. In light of the new evidence regarding the lack of miR-143/145 functionality in intestinal epithelial cells, one may hypothesize that miR-143/145 may function as a surrogate marker for tumor infiltration with mesenchymal cells rather than repression of target genes, such as *KRAS*. Thus it would be interesting to see an expression analysis of miR-143/145 using *in situ* hybridization of CRC tissue microarrays, and examine co-localization with their validated target genes, such *KRAS, NKRAS, CTNNG1, ERK* and *KLF5*.

The *LEF/TCF* complex activates numerous pro-survival signaling cascades and some miRNAs may be under direct *LEF/TCF* control and/or being regulated by downstream effectors [119, 120]. By using either a bioinformatics approach to discover miRNA transcription start sites within close proximity to *TCF4* chromatin occupancy sites [120] or profiling miRNA expression in CRC cells with disrupted *CTNNB1*/*TCF4* activity followed by integration of *TCF4* chromatin data [119], roughly 30 *TCF4*-responsive miRNAs have been identified. However, only miR-23b was found in both studies.

The *TCF/LEF*–dependent transcriptional activity is most likely regulated by miR-34 family members, since the UTRs of multiple WNT pathway genes contain binding sites for this family of miRNAs [27, 121-123]. MiR-34, activated by *TP53,* inhibited activity of the *TCF/LEF* complex and linked the canonical WNT pathway with *TP53* activity [122] (Figure 4). Loss of *TP53* function resulted in increased activity of WNT signaling cascade and promoted the Snail-dependent EMT program [122]. Interestingly, UTRs of oncogenes, including *CTNNB1*, are often shortened in cancer cells as a consequence of alternative cleavage, leading to loss of miR-34 mediated regulation [121, 122]. Furthermore *MYC,* induced by *WNT,* upregulated the oncogenic miR-17-92 miRNA cluster that encode miRNAs, directly targeting the key effector of TGFR signaling pathway, *SMAD4* [124].

### Activation of EGFR signaling

The epidermal growth factor receptor (*EGFR*) signaling pathway coordinates a variety of cellular activities [125]. The cancers with mutated *EGFR* have been suggested to have an “oncogene addiction”, serving as the basis for EGFR targeted therapies. However, the clinical benefit from EGFR-targeted therapies has so far been rather limited in CRC. Activating *KRAS* mutations, functioning downstream of EGFR, have been reported in 30–60% of CRCs [126]. These patients do not benefit from anti-EGFR treatment. Furthermore, among patients with wild type *KRAS*, a clinical benefit is only seen in 10 – 20% [127, 128]. Activating mutations of *BRAF*, as well as of *PIK3CA* and loss of *PTEN* are also associated with lack of response to anti-EGFR therapy. Nevertheless, they cannot alone explain the low success rate of the treatment. In search of additive deregulation mechanisms, multiple studies have focused on miRNA association with *KRAS* and *PTEN*, while other genes of the EFGR pathway have received less attention.

The *KRAS* gene is directly targeted by the let-7 miRNA family [129]. A SNP, present in the *KRAS* 3′UTR, disturbs the let-7 binding site, resulting in increased *KRAS* mRNA levels. *KRAS*-let-7 SNP variant carriers were shown to have an increased presentation of advanced colon cancer [130], however, conflicting results are published regarding the response to chemotherapy and CRC outcome [130-133]. Recently, the *KRAS*-let-7 SNP variant was reported to correctly predict a clinical response to anti-EGFR therapy in a large cohort of patients with metastatic CRC [134]. Other miRNAs have been shown to repress *KRAS*, in particular, miR-143 and miR-145 have received a lot of attention [135]. Restoring miR-143/145 in colon cancer cells decreased proliferation, migration and chemoresistance, however, in light of the recent results by Chivukala and colleagues [105], the biological relevance of miR-143/145 in epithelial malignancies needs re-examination. Other studies revealed that the star strand of mir-18a and miR-4689 function as potential tumor suppressors by targeting *KRAS* [136, 137]. In addition, miR-31 activated the RAS signaling pathway by repressing *RASA1* [138].

Multiple miRNAs have been shown to target the PIK3/AKT hub downstream in the *EGFR* signaling pathway [139]. Mutations in the *PIK3CA* coding sequence (exons 9 and 20) and a novel 3′UTR mutation, reducing binding affinity for miR-520a and miR-525a, were associated to increased sensitivity to saracatinib, a Src kinase inhibitor [140]. This mutation could be used as predictive marker for saracatinib therapy and may improve effectiveness of this drug. Importantly, a phase II study of saracatinib in unselected metastatic CRC patients showed no improvement in progression-free survival [141]. MiR-126, which expression is frequently lost in CRC, modulated the activity of PI3K at the level of signal initiation by limiting *PIK3R2* levels in normal colon epithelium [142], while miR-30a, which expression is also reduced in metastatic CRC, has been reported to target *PIK3CD* [143].

*PTEN*, a central negative regulator of the PIK3/AKT, is inactivated by mutations and/or deletions in many primary and metastatic human cancers [144, 145]. In CRC the *PTEN* transcript has been shown to be targeted by multiple miRNAs (Figure 4C), including miR-19 [146], miR-21 [147], miR-32 [148] and miR-92-1-5p [149]. Despite the fact that *PTEN* was most frequently reported to be repressed by miR-21, miR-21 and *PTEN* expression showed no association in TCGA CRC patient cohort [44]. Upon enforced miR-26a in *APC*^min/+^ mice, a model known to be sensitive to PTEN dosage [19], potent reduction of tumor number and size was observed, suggesting a therapeutic potential of miR-26a.

### Impairment of TP53 function

The tumor suppressor *TP53* responds to diverse stress signals by coordinating specific cellular responses, including cell cycle arrest, senescence, apoptosis, invasion and metastasis, as well as cell–cell communication within the tumor microenvironment [150]. The importance of *TP53* in tumor suppression is unequivocal, as shown by its inactivation in more than half of all sporadic human cancers, including CRC [151]. Wild-type *TP53* encodes a sequence-specific activator which exerts its function through transcriptional regulation of protein-coding genes to initiate cellular responses. Many genes have been identified to contain TP53-responsive elements and be induced upon TP53 activation. Despite that global bioinformatic sequence analysis suggested that up to 46% of the miRNA putative promoters contain a potential *TP53*-binding site [152], only approximately 50 miRNAs have been experimentally validated to be under transcriptional control of *TP53.* Among them, miRNAs, let-7i, miR-20a, miR-21, miR-25, miR-34a/b/c, miR-145, miR-181b, miR-183, miR-195, miR-215, and miR-451 have been reported by two or more studies. MiR-34a and other members of the miR-34 family were identified as TP53-inducible miRNAs in several cancer types [153, 154]. The miR-34 family comprises three miRNAs; miR-34a is encoded by its own transcript, whereas miR-34b and miR-34c share a common primary transcript. MiR-34a is expressed at high levels in CRC patients, while miR-34b/c expression is not detectable. An epigenetic silencing of miR-34b/c has been reported in CRC cell lines [155]. Silencing of miR-34 by aberrant CpG methylation was dominant over its transactivation by TP53 after DNA damage [102], impairing the tumor suppressive role of *TP53*, particularly for cancer cells not exhibiting *TP53* mutation. Finally, miR-34a was found to activate *TP53* by inhibiting *SIRT1*, the major regulator of *TP53* [156, 157] suggesting a positive feedback loop between *TP53* and miR-34a.

Interestingly, miR-192 and miR-215 may act as effectors as well as regulators of *TP53* by suppressing tumorigenesis through *CDKN1A* [158]. The fact that miR-192 and miR-215 were reduced in CRC while being highly expressed in normal colon further supports the idea that these miRNAs carry out a tumor suppressive function. Among other *TP53* responsible miRNAs, miR-200 cluster family members play a critical role in metastasis, as previously discussed.

*TP53* itself is subjected to miRNA induced regulation and miR-125b has been validated as such a negative regulator in CRC [159], which upon high expression is associated with tumor initiation, progression, invasiveness, and poor prognosis. Intriguingly, in relation to miR-125 expression levels, no significant difference was observed between patients with wild type *TP53* or *TP53* mutation [159]. Other miRNAs have been reported to control *TP53* indirectly, by regulating *MDM2 (*miR-339-5p) [160] and *RFFL* (miR-133a) [138].

Although, several of the examples described above illustrate importance of miRNA induced regulation, we have only begun to understand the role of the complex regulatory loops in the *TP53-*miRNA network. It also should be noted that besides the expression regulation of miRNA-coding genes, *TP53* may directly affect the processing of miRNAs [161].

### Inactivation of TGF-β signaling

A wide spectrum of cellular functions such as proliferation, apoptosis, differentiation, migration and interactions with the microenvironment are regulated by TGF-β family members. The TGF-β pathway is activated when the ligand binds to TGFBR2 at the cell surface and TGFBR1 is recruited and phosphorylated. TGFBR2 is pivotal to trigger the signaling pathway, and signals through SMADs or non-SMAD pathways [162]. Tumor cells often escape from the anti-proliferative effects of *TGF*-β by mutational inactivation of its components. Mutations in *TGFBR2* are estimated to occur in approximately 90% of MSI and in 15% of microsatellite stable (MSS) CRCs [163]. Germline mutations in *SMAD4* and *BMPR1A* are found in patients with familial juvenile polyposis, an autosomal dominant condition, and associated with increased risk of CRC [164]. Multiple miRNAs have been confirmed to regulate *TGFBR2*, such as miR-17-5p, miR-20a, miR-21, miR-23b, miR-106a and miR-301a (Figure 3C). The miR-21-*TGFBR2* pair had also significant negative associations in TCGA CRC patients, confirming this regulatory node in large patient series. Notably, miR-21, activated by the WNT signaling pathway, regulated stemness by modulating *TGFBR2* signaling, which induced chemoresistance in animal models [165].

The oncogenic miR-17-92 cluster, regulated by *MYC*, has executed *TGF*-β responses by targeting *TGFBR2* and *SMAD4* [124], interconnecting the TGF-β and WNT signaling pathways. On the other hand, overexpression of *MYC* and DNA copy number gain of the miR-17-92 locus on 13q31 were frequently seen in non–MSI tumors, indicating that miR-17-92 induced repression could be an alternative mechanism for inactivation of the *TGF*-β pathway. Interestingly, the miR-106a/363 and miR-106b/25 clusters encode paralogous miRNAs to those in the miR-17-92 cluster and, consequently, target some of the same genes and pathways including *TGFBR2* and *SMAD2*/*SMAD4* [124, 166]. Furthermore, miR-130a, miR-301a, and miR-454, frequently upregulated in CRC tissues, have been shown to target *SMAD4* resulting in enhanced cell proliferation and migration [167]. The activation of *SMAD7*, a negative regulator of the TGF-β signaling pathway, promoted EMT and metastasis as a result of reduced miR-25 expression [168]. The non-SMAD TGF-β pathways include various branches of MAP/PIK3/AKT and RHO-like GTPase signaling. Alterations in Rho GTPase gene expression levels, rather than constitutive mutations, are often associated with tumorigenesis and cancer progression [169]. The reduced expression may be caused, at least in part, by the matched 3′UTRs repression by miRNAs. For example, miR-185 inhibited the expression of *RHOA* and *CDC42* resulting in reduced proliferation and induced G1 cell cycle arrest and apoptosis [170]. *CDC42* has also been validated as a target for miR-137 [171], while *RHOB* and *RHOBTB1* have been repressed by miR-21 and miR-31, respectively. This links regulation of RHO GTPase gene expression with WNT and EGFR signaling pathways.

## CONCLUDING REMARKS

The field of miRNA research has grown tremendously due to methodological developments and discoveries of new cellular roles of miRNAs. They seem to play a role at every stage of the tumor development, however, their necessity in driving of tumor growth still needs to be proven. So far, metastasis stands out as the process most tightly regulated by miRNAs. Despite the fact that miRNAs are known to have multiple targets, pair-wise miRNA-target validation has dominated target investigations in CRC as summarized and discussed in this review. As a result, our understanding of the regulatory miRNA networks and how family miRNAs could compensate for each others' functions remains limited.

The identified altered miRNA expression in CRC has also raised exciting opportunities for clinical applications and we have here reviewed several miRNAs with potential as biomarkers for diagnosis and prognosis. However, the challenge remains to validate miRNA biomarkers in large, independent patient cohorts. Evidence that miRNAs contribute to several aspects of tumorigenesis suggests that inhibition of highly expressed miRNAs, or replacement of miRNAs with reduced expression, could become treatment strategies. On the other hand, the ability of miRNAs to regulate multiple targets might increase the efficacy of miRNA-based drugs as well as lead to undesirable side effects. Understanding the molecular and cellular pathways that are controlled by miRNAs, as illustrated for the main pathways of CRC development, may facilitate development of miRNA-therapeutics.

Taken together, these directions are innovative and promising. Nonetheless, the clinical impact for CRC of miRNAs identified in the proof–of-concept studies in cell lines, animal models and small patient cohorts has to be confirmed in carefully designed clinical studies.

## SUPPLEMENTARY MATERIAL FIGURE AND TABLE





## References

[1] Ferlay J., Steliarova-Foucher E., Lortet-Tieulent J., Rosso S., Coebergh J. W., Comber H., Forman D., Bray F (2013). Cancer incidence and mortality patterns in Europe: Estimates for 40 countries in 2012. Eur J Cancer.

[2] Stewart B. W., Wild C. P. (2014). World Cancer Report.

[3] Friedman R. C., Farh K. K., Burge C. B., Bartel D. P. (2009). Most mammalian mRNAs are conserved targets of microRNAs. Genome Res.

[4] Kozomara A., Griffiths-Jones S. (2014). miRBase: annotating high confidence microRNAs using deep sequencing data. Nucleic Acids Research.

[5] Mestdagh P., Hartmann N., Baeriswyl L., Andreasen D., Bernard N., Chen C., Cheo D., D'andrade P., Demayo M., Dennis L., Derveaux S., Feng Y., Fulmer-Smentek S. (2014). Evaluation of quantitative miRNA expression platforms in the microRNA quality control (miRQC) study. Nat Methods.

[6] Brennecke J., Hipfner D. R., Stark A., Russell R. B., Cohen S. M. (2003). bantam Encodes a Developmentally Regulated microRNA that Controls Cell Proliferation and Regulates the Proapoptotic Gene hid in Drosophila. Cell.

[7] Chen Ch., Li L., Lodish H. F., Bartel D.P. (2004). MicroRNAs Modulate Hematopoietic Lineage Differentiation. Science.

[8] Lee R. C., Feinbaum R. L., Ambros V. (1993). The C. elegans heterochronic gene lin-4 encodes small RNAs with antisense complementarity to lin-14. Cell.

[9] Pasquinelli A. E., Reinhart B. J., Slack F., Martindale M. Q., Kuroda M. I., Maller B., Hayward D. C., Ball E. E., Degnan B., Muller P., Spring J., Srinivasan A., Fishman M. (2000). Conservation of the sequence and temporal expression of let-7 heterochronic regulatory RNA. Nature.

[10] Calin G. A., Dumitru C. D., Shimizu M., Bichi R., Zupo S., Noch E., Aldler H., Rattan S., Keating M., Rai K., Rassenti L., Kipps T., Negrini M. (2002). Frequent deletions and down-regulation of microRNA genes miR15 and miR16 at 13q14 in chronic lymphocytic leukemia. Proc Natl Acad Sci U S A.

[11] Michael M. Z., Connor Sm O', Van Holst Pellekaan N. G., Young G. P., James R. J. (2003). Reduced accumulation of specific microRNAs in colorectal neoplasia. Mol Cancer Res.

[12] Takamizawa J., Konishi H., Yanagisawa K., Tomida S., Osada H., Endoh H., Harano T., Yatabe Y., Nagino M., Nimura Y., Mitsudomi T., Takahashi T. (2004). Reduced expression of the let-7 microRNAs in human lung cancers in association with shortened postoperative survival. Cancer Res.

[13] O'donnell K. A., Wentzel E. A., Zeller K. I., Dang C. V., Mendell J. T. (2005). c-Myc-regulated microRNAs modulate E2F1 expression. Nature.

[14] Ma L., Teruya-Feldstein J., Weinberg R. A. (2007). Tumour invasion and metastasis initiated by microRNA-10b in breast cancer. Nature.

[15] Meiri E., Mueller W. C., Rosenwald S., Zepeniuk M., Klinke E., Edmonston T. B., Werner M., Lass U., Barshack I., Feinmesser M., Huszar M., Fogt F., Ashkenazi K. (2012). A second-generation microRNA-based assay for diagnosing tumor tissue origin. Oncologist.

[16] (2015). A Multicenter Phase I Study of MRX34, MicroRNA miR-RX34 Liposomal Injection. http://Clinicaltrials.Gov.

[17] Ambros V., Bartel B., Bartel D. P., Burge C. B., Carrington J. C., Chen X., Dreyfuss G., Eddy S. R., Griffiths-Jones S., Marshall M., Matzke M., Ruvkun G., Tuschl T (2003). A uniform system for microRNA annotation. RNA.

[18] Spizzo R., Nicoloso M. S., Croce C. M., Calin G. A. (2009). SnapShot: MicroRNAs in Cancer. Cell.

[19] Zeitels L. R., Acharya A., Shi G., Chivukula D., Chivukula R. R., Anandam J. L., Abdelnaby A. A., Balch G. C., Mansour J. C., Yopp A. C., Richardson J. A., Mendell J. T. (2014). Tumor suppression by miR-26 overrides potential oncogenic activity in intestinal tumorigenesis. Genes Dev.

[20] Batlle E., Sancho E., Franci C., Dominguez D., Monfar M., Baulida J., Garcia De Herreros A. (2000). The transcription factor snail is a repressor of E-cadherin gene expression in epithelial tumour cells. Nat Cell Biol.

[21] Eger A., Aigner K., Sonderegger S., Dampier B., Oehler S., Schreiber M., Berx G., Cano A., Beug H., Foisner R. (2005). DeltaEF1 is a transcriptional repressor of E-cadherin and regulates epithelial plasticity in breast cancer cells. Oncogene.

[22] Perl A. K., Wilgenbus P., Dahl U., Semb H., Christofori G (1998). A causal role for E-cadherin in the transition from adenoma to carcinoma. Nature.

[23] Siemens H., Jackstadt R., Hunten S., Kaller M., Menssen A., Gotz U., Hermeking H. (2011). miR-34 and SNAIL form a double-negative feedback loop to regulate epithelial-mesenchymal transitions. Cell Cycle.

[24] Roy S., Levi E., Majumdar A. P., Sarkar F.H. (2012). Expression of miR-34 is lost in colon cancer which can be re-expressed by a novel agent CDF. J Hematol Oncol.

[25] Hahn S., Jackstadt R., Siemens H., Hunten S., Hermeking H. (2013). SNAIL, miR-34a feed-forward regulation of ZNF281/ZBP99 promotes epithelial-mesenchymal transition. EMBO J.

[26] Rokavec M., Oner M. G., Li H., Jackstadt R., Iang L., Lodygin D., Kaller M., Horst D., Ziegler P. K., Schwitalla S., Slotta-Huspenina J., Bader F. G., Greten F. R. (2014). IL-6R/STAT3/miR-34a feedback loop promotes EMT-mediated colorectal cancer invasion and metastasis. J Clin Invest.

[27] Siemens H., Neumann J., Jackstadt R., Mansmann U., Horst D., Kirchner T., Hermeking H. (2013). Detection of miR-34a promoter methylation in combination with elevated expression of c-Met and beta-catenin predicts distant metastasis of colon cancer. Clin Cancer Res.

[28] Gao J., Li N., Dong Y., Li S., Xu L., Li X., Li Y., Li Z., Ng S. S., Sung J. J., Shen L., Yu J (2015). miR-34a-5p suppresses colorectal cancer metastasis and predicts recurrence in patients with stage II/III colorectal cancer. Oncogene.

[29] Li X., Zhao H., Zhou X., Song L. (2015). Inhibition of lactate dehydrogenase A by microRNA-34a resensitizes colon cancer cells to 5-fluorouracil. Mol Med Rep.

[30] (2015). Mirna Therapeutics Interim Phase 1 Data. http://www.mirnarx.com/.

[31] Burk U., Schubert J., Wellner U., Schmalhofer O., Vincan E., Spaderna S., Brabletz T. (2008). A reciprocal repression between ZEB1 and members of the miR-200 family promotes EMT and invasion in cancer cells. EMBO Rep.

[32] Wellner U., Schubert J., Burk U. C., Schmalhofer O., Zhu F., Sonntag A., Waldvogel B., Vannier C., Darling D., Zur Hausen A., Brunton V. G., Morton J., Sansom O. (2009). The EMT-activator ZEB1 promotes tumorigenicity by repressing stemness-inhibiting microRNAs. Nat Cell Biol.

[33] Chen M. L., Liang L. S., Wang X. K. (2012). miR-200c inhibits invasion and migration in human colon cancer cells SW480/620 by targeting ZEB1. Clin Exp Metastasis.

[34] Davalos V., Moutinho C., Villanueva A., Boque R., Silva P., Carneiro F., Esteller M. (2012). Dynamic epigenetic regulation of the microRNA-200 family mediates epithelial and mesenchymal transitions in human tumorigenesis. Oncogene.

[35] Paterson E. L., Kazenwadel J., Bert A. G., Khew-Goodall Y., Ruszkiewicz A., Goodall G. J. (2013). Down-regulation of the miRNA-200 family at the invasive front of colorectal cancers with degraded basement membrane indicates EMT is involved in cancer progression. Neoplasia.

[36] Hur K., Toiyama Y., Takahashi M., Balaguer F., Nagasaka T., Koike J., Hemmi H., Koi M., Boland C.R., Goel A. (2013). MicroRNA-200c modulates epithelial-to-mesenchymal transition (EMT) in human colorectal cancer metastasis. Gut.

[37] Sui H., Cai G. X., Pan S. F., Deng W. L., Wang Y. W., Chen Z. S., Cai S. J., Zhu H. R., Li Q (2014). miR200c attenuates P-gp-mediated MDR and metastasis by targeting JNK2/c-Jun signaling pathway in colorectal cancer. Mol Cancer Ther.

[38] Kahlert C., Klupp F., Brand K., Lasitschka F., Diederichs S., Kirchberg J., Rahbari N., Dutta S., Bork U., Fritzmann J., Reissfelder C., Koch M., Weitz J. (2011). Invasion front-specific expression and prognostic significance of microRNA in colorectal liver metastases. Cancer Sci.

[39] Lu M. H., Huang C. C., Pan M. R., Chen H. H., Hung W. C. (2012). Prospero homeobox 1 promotes epithelial-mesenchymal transition in colon cancer cells by inhibiting E-cadherin via miR-9. Clin Cancer Res.

[40] Cekaite L., Rantala J. K., Bruun J., Guriby M., Agesen T. H., Danielsen S. A., Lind G. E., Nesbakken A., Kallioniemi O., Lothe R. A., Skotheim R. I. (2012). MiR-9, -31, and -182 deregulation promote proliferation and tumor cell survival in colon cancer. Neoplasia.

[41] Cancer Genome Atlas Network (2012). Comprehensive molecular characterization of human colon and rectal cancer. Nature.

[42] Ma F., Song H., Guo B., Zhang Y., Zheng Y., Lin C., Wu Y., Guan G., Sha R., Zhou Q., Wang D., Zhou X., Li J. (2015). MiR-361-5p inhibits colorectal and gastric cancer growth and metastasis by targeting staphylococcal nuclease domain containing-1. Oncotarget.

[43] Zhang H., Hao Y., Yang J., Zhou Y., Li J., Yin S., Sun C., Ma M., Huang Y., Xi J.J. (2011). Genome-wide functional screening of miR-23b as a pleiotropic modulator suppressing cancer metastasis. Nat Commun.

[44] Okamoto K., Ishiguro T., Midorikawa Y., Ohata H., Izumiya M., Tsuchiya N., Sato A., Sakai H., Nakagama H. (2012). miR-493 induction during carcinogenesis blocks metastatic settlement of colon cancer cells in liver. EMBO J.

[45] Loo J. M., Scherl A., Nguyen A., Man F. Y., Weinberg E., Zeng Z., Saltz L., Paty P. B., Tavazoie S. F. (2015). Extracellular metabolic energetics can promote cancer progression. Cell.

[46] Li Y., Zhang Z. (2014). Potential microRNA-mediated oncogenic intercellular communication revealed by pan-cancer analysis. Sci Rep.

[47] Rosetta Genomics (2015). http://www.rosettagenomics.com/.

[48] Nosho K., Igarashi H., Nojima M., Ito M., Maruyama R., Yoshii S., Naito T., Sukawa Y., Mikami M., Sumioka W., Yamamoto E., Kurokawa S., Adachi Y. (2014). Association of microRNA-31 with BRAF mutation, colorectal cancer survival, serrated pathway. Carcinogenesis.

[49] Wang S., Wang L., Bayaxi N., Li J., Verhaegh W., Janevski A., Varadan V., Ren Y., Merkle D., Meng X., Gao X., Wang H., Ren J. (2013). A microRNA panel to discriminate carcinomas from high-grade intraepithelial neoplasms in colonoscopy biopsy tissue. Gut.

[50] Weissmann-Brenner A., Kushnir M., Lithwick Yanai G., Aharonov R., Gibori H., Purim O., Kundel Y., Morgenstern S., Halperin M., Niv Y., Brenner B. (2012). Tumor microRNA-29a expression and the risk of recurrence in stage II colon cancer. Int J Oncol.

[51] Van De Velde C. J., Boelens P. G., Borras J. M., Coebergh J. W., Cervantes A., Blomqvist L., Beets-Tan R. G., Van Den Broek C. B., Brown G., Van Cutsem E., Espin E., Haustermans K., Glimelius B. (2014). EURECCA colorectal: multidisciplinary management: European consensus conference colon & rectum. Eur J Cancer.

[52] Zhang J. X., Song W., Chen Z. H., Wei J. H., Liao Y. J., Lei J., Hu M., Chen G. Z., Liao B., Lu J., Zhao H. W., Chen W., He Y. L. (2013). Prognostic and predictive value of a microRNA signature in stage II colon cancer: a microRNA expression analysis. Lancet Oncol.

[53] Kang W. K., Lee J. K., Oh S. T., Lee S. H., Jung C. K. (2015). Stromal expression of miR-21 in T3-4a colorectal cancer is an independent predictor of early tumor relapse. BMC Gastroenterol.

[54] Diaz T., Tejero R., Moreno I., Ferrer G., Cordeiro A., Artells R., Navarro A., Hernandez R., Tapia G., Monzo M. (2014). Role of miR-200 family members in survival of colorectal cancer patients treated with fluoropyrimidines. J Surg Oncol.

[55] Liu H., Du L., Wen Z., Yang Y., Li J., Wang L., Zhang X., Liu Y., Dong Z., Li W., Zheng G., Wang C. (2013). Up-regulation of miR-182 expression in colorectal cancer tissues and its prognostic value. Int J Colorectal Dis.

[56] Huynh C., Segura M. F., Gaziel-Sovran A., Menendez S., Darvishian F., Chiriboga L., Levin B., Meruelo D., Osman I., Zavadil J., Marcusson E. G., Hernando E (2011). Efficient in vivo microRNA targeting of liver metastasis. Oncogene.

[57] Ma Y., Zhang P., Wang F., Zhang H., Yang J., Peng J., Liu W., Qin H. (2012). miR-150 as a potential biomarker associated with prognosis and therapeutic outcome in colorectal cancer. Gut.

[58] Ma W., Yu J., Qi X., Liang L., Zhang Y., Ding Y., Lin X., Li G., Ding Y. (2015). Radiation-induced microRNA-622 causes radioresistance in colorectal cancer cells by down-regulating Rb. Oncotarget.

[59] Cappuzzo F., Sacconi A., Landi L., Ludovini V., Biagioni F., D'incecco A., Capodanno A., Salvini J., Corgna E., Cupini S., Barbara C., Fontanini G., Crino L. (2014). MicroRNA signature in metastatic colorectal cancer patients treated with anti-EGFR monoclonal antibodies. Clin Colorectal Cancer.

[60] Pichler M., Winter E., Ress A. L., Bauernhofer T., Gerger A., Kiesslich T., Lax S., Samonigg H., Hoefler G. (2014). miR-181a is associated with poor clinical outcome in patients with colorectal cancer treated with EGFR inhibitor. J Clin Pathol.

[61] Suto T., Yokobori T., Yajima R., Morita H., Fujii T., Yamaguchi S., Altan B., Tsutsumi S., Asao T., Kuwano H. (2015). MicroRNA-7 expression in colorectal cancer is associated with poor prognosis and regulates cetuximab sensitivity via EGFR regulation. Carcinogenesis.

[62] Kanaan Z., Rai S. N., Eichenberger M. R., Roberts H., Keskey B., Pan J., Galandiuk S. (2012). Plasma miR-21: a potential diagnostic marker of colorectal cancer. Ann Surg.

[63] Monzo M., Martinez-Rodenas F., Moreno I., Navarro A., Santasusagna S., Macias I., Munoz C., Tejero R., Hernandez R. (2015). Differential MIR-21 expression in plasma from mesenteric versus peripheral veins: an observational study of disease-free survival in surgically resected colon cancer patients. Medicine (Baltimore).

[64] Pu X. X., Huang G. L., Guo H. Q., Guo C. C., Li H., Ye S., Ling S., Jiang L., Tian Y., Lin T. Y. (2010). Circulating miR-221 directly amplified from plasma is a potential diagnostic and prognostic marker of colorectal cancer and is correlated with p53 expression. J Gastroenterol Hepatol.

[65] Shan L., Ji Q., Cheng G., Xia J., Liu D., Wu C., Zhu B., Ding Y. (2015). Diagnostic value of circulating miR-21 for colorectal cancer: a meta-analysis. Cancer Biomark.

[66] Ng E. K., Chong W. W., Jin H., Lam E. K., Shin V. Y., Yu J., Poon T. C., Ng S. S., Sung J. J. (2009). Differential expression of microRNAs in plasma of patients with colorectal cancer: a potential marker for colorectal cancer screening. Gut.

[67] Wang S., Xiang J., Li Z., Lu S., Hu J., Gao X., Yu L., Wang L., Wang J., Wu Y., Chen Z., Zhu H. (2013). A plasma microRNA panel for early detection of colorectal cancer. Int J Cancer.

[68] Yuan D., Li K., Zhu K., Yan R., Dang C. (2015). Plasma miR-183 predicts recurrence and prognosis in patients with colorectal cancer. Cancer Biol Ther.

[69] Perilli L., Vicentini C., Agostini M., Pizzini S., Pizzi M., D'angelo E., Bortoluzzi S., Mandruzzato S., Mammano E., Rugge M., Nitti D., Scarpa A., Fassan M. (2014). Circulating miR-182 is a biomarker of colorectal adenocarcinoma progression. Oncotarget.

[70] Kjersem J. B., Ikdahl T., Lingjaerde O. C., Guren T., Tveit K. M., Kure E. H. (2014). Plasma microRNAs predicting clinical outcome in metastatic colorectal cancer patients receiving first-line oxaliplatin-based treatment. Mol Oncol.

[71] Koga Y., Yamazaki N., Yamamoto Y., Yamamoto S., Saito N., Kakugawa Y., Otake Y., Matsumoto M., Matsumura Y. (2013). Fecal miR-106a is a useful marker for colorectal cancer patients with false-negative results in immunochemical fecal occult blood test. Cancer Epidemiol Biomarkers Prev.

[72] Koga Y., Yasunaga M., Takahashi A., Kuroda J., Moriya Y., Akasu T., Fujita S., Yamamoto S., Baba H., Matsumura Y. (2010). MicroRNA expression profiling of exfoliated colonocytes isolated from feces for colorectal cancer screening. Cancer Prev Res (Phila).

[73] Zhang J., Fei B., Wang Q., Song M., Yin Y., Zhang B., Ni S., Guo W., Bian Z., Quan C., Liu Z., Wang Y., Yu J. (2014). MicroRNA-638 inhibits cell proliferation, invasion and regulates cell cycle by targeting tetraspanin 1 in human colorectal carcinoma. Oncotarget.

[74] Inoue A., Yamamoto H., Uemura M., Nishimura J., Hata T., Takemasa I., Ikenaga M., Ikeda M., Murata K., Mizushima T., Doki Y., Mori M. (2014). MicroRNA-29b is a Novel Prognostic Marker in Colorectal Cancer. Ann Surg Oncol.

[75] Nakano H., Miyazawa T., Kinoshita K., Yamada Y., Yoshida T. (2010). Functional screening identifies a microRNA, miR-491 that induces apoptosis by targeting Bcl-X(L) in colorectal cancer cells. Int J Cancer.

[76] Christensen L. L., Holm A., Rantala J., Kallioniemi O., Rasmussen M. H., Ostenfeld M. S., Dagnaes-Hansen F., Oster B., Schepeler T., Tobiasen H., Thorsen K., Sieber O. M., Gibbs P. (2014). Functional screening identifies miRNAs influencing apoptosis and proliferation in colorectal cancer. PLoS One.

[77] Venkatachalam R., Verwiel E. T., Kamping E. J., Hoenselaar E., Gorgens H., Schackert H. K., Van Krieken J. H., Ligtenberg M. J., Hoogerbrugge N., Van Kessel A. G., Kuiper R. P. (2011). Identification of candidate predisposing copy number variants in familial and early-onset colorectal cancer patients. Int J Cancer.

[78] Rapti S. M., Kontos C. K., Papadopoulos I. N., Scorilas A (2014). Enhanced miR-182 transcription is a predictor of poor overall survival in colorectal adenocarcinoma patients. Clin Chem Lab Med.

[79] Sarver A. L., French A. J., Borralho P. M., Thayanithy V., Oberg A. L., Silverstein K. A., Morlan B. W., Riska S. M., Boardman L. A., Cunningham J. M., Subramanian S., Wang L., Smyrk T. C. (2009). Human colon cancer profiles show differential microRNA expression depending on mismatch repair status and are characteristic of undifferentiated proliferative states. BMC Cancer.

[80] Pizzini S., Bisognin A., Mandruzzato S., Biasiolo M., Facciolli A., Perilli L., Rossi E., Esposito G., Rugge M., Pilati P., Mocellin S., Nitti D., Bortoluzzi S. (2013). Impact of microRNAs on regulatory networks and pathways in human colorectal carcinogenesis and development of metastasis. BMC Genomics.

[81] Yang M. H., Yu J., Jiang D. M., Li W. L., Wang S., Ding Y. Q. (2014). microRNA-182 targets special AT-rich sequence-binding protein 2 to promote colorectal cancer proliferation and metastasis. J Transl Med.

[82] Amodeo V., Bazan V., Fanale D., Insalaco L., Caruso S., Cicero G., Bronte G., Rolfo C., Santini D., Russo A. (2013). Effects of anti-miR-182 on TSP-1 expression in human colon cancer cells: there is a sense in antisense?. Expert Opin Ther Targets.

[83] Li L., Sarver A. L., Khatri R., Hajeri P. B., Kamenev I., French A. J., Thibodeau S. N., Steer C. J., Subramanian S (2014). Sequential expression of miR-182 and miR-503 cooperatively targets FBXW7, contributing to the malignant transformation of colon adenoma to adenocarcinoma. J Pathol.

[84] Lengauer C., Kinzler K.W., Vogelstein B. (1997). Genetic instability in colorectal cancers. Nature.

[85] Shibata D., Peinado M. A., Ionov Y., Malkhosyan S., Perucho M (1994). Genomic instability in repeated sequences is an early somatic event in colorectal tumorigenesis that persists after transformation. Nat Genet.

[86] Boland C.R., Goel A. (2010). Microsatellite instability in colorectal cancer. Gastroenterology.

[87] Toyota M., Ahuja N., Ohe-Toyota M., Herman J. G., Baylin S. B., Issa J. P. (1999). CpG island methylator phenotype in colorectal cancer. Proc Natl Acad Sci U S A.

[88] Sveen A., Agesen T. H., Nesbakken A., Rognum T. O., Lothe R. A., Skotheim R. I. (2011). Transcriptome instability in colorectal cancer identified by exon microarray analyses: Associations with splicing factor expression levels and patient survival. Genome Med.

[89] Lanza G., Ferracin M., Gafa R., Veronese A., Spizzo R., Pichiorri F., Liu C. G., Calin G. A., Croce C. M., Negrini M. (2007). mRNA/microRNA gene expression profile in microsatellite unstable colorectal cancer. Mol Cancer.

[90] Earle J. S., Luthra R., Romans A., Abraham R., Ensor J., Yao H., Hamilton S. R. (2010). Association of microRNA expression with microsatellite instability status in colorectal adenocarcinoma. J Mol Diagn.

[91] Valeri N., Gasparini P., Fabbri M., Braconi C., Veronese A., Lovat F., Adair B., Vannini I., Fanini F., Bottoni A., Costinean S., Sandhu S. K., Nuovo G. J. (2010). Modulation of mismatch repair and genomic stability by miR-155. Proc Natl Acad Sci U S A.

[92] Balaguer F., Moreira L., Lozano J. J., Link A., Ramirez G., Shen Y., Cuatrecasas M., Arnold M., Meltzer S. J., Syngal S., Stoffel E., Jover R., Llor X. (2011). Colorectal cancers with microsatellite instability display unique miRNA profiles. Clin Cancer Res.

[93] Donehower L. A., Creighton C. J., Schultz N., Shinbrot E., Chang K., Gunaratne P. H., Muzny D., Sander C., Hamilton S. R., Gibbs R. A., Wheeler D (2013). MLH1-silenced and non-silenced subgroups of hypermutated colorectal carcinomas have distinct mutational landscapes. J Pathol.

[94] Oster B., Linnet L., Christensen L. L., Thorsen K., Ongen H., Dermitzakis E. T., Sandoval J., Moran S., Esteller M., Hansen T. F., Lamy P., Laurberg S., Group Colofol Steering (2013). Non-CpG island promoter hypomethylation and miR-149 regulate the expression of SRPX2 in colorectal cancer. Int J Cancer.

[95] Svrcek M., El-Murr N., Wanherdrick K., Dumont S., Beaugerie L., Cosnes J., Colombel J. F., Tiret E., Flejou J. F., Lesuffleur T., Duval A. (2013). Overexpression of microRNAs-155 and 21 targeting mismatch repair proteins in inflammatory bowel diseases. Carcinogenesis.

[96] Li X., Li X., Liao D., Wang X., Wu Z., Nie J., Bai M., Fu X., Mei Q., Han W. (2015). Elevated microRNA-23a Expression Enhances the Chemoresistance of Colorectal Cancer Cells with Microsatellite Instability to 5-Fluorouracil by Directly Targeting ABCF1. Curr Protein Pept Sci.

[97] Valeri N., Gasparini P., Braconi C., Paone A., Lovat F., Fabbri M., Sumani K. M., Alder H., Amadori D., Patel T., Nuovo G. J., Fishel R., Croce C. M. (2010). MicroRNA-21 induces resistance to 5-fluorouracil by down-regulating human DNA MutS homolog 2 (hMSH2). Proc Natl Acad Sci U S A.

[98] El-Murr N., Abidi Z., Wanherdrick K., Svrcek M., Gaub M. P., Flejou J. F., Hamelin R., Duval A., Lesuffleur T (2012). MiRNA genes constitute new targets for microsatellite instability in colorectal cancer. PLoS One.

[99] Calin G. A., Sevignani C., Dumitru C. D., Hyslop T., Noch E., Yendamuri S., Shimizu M., Rattan S., Bullrich F., Negrini M., Croce C. M. (2004). Human microRNA genes are frequently located at fragile sites, genomic regions involved in cancers. Proc Natl Acad Sci U S A.

[100] Diosdado B., Van De Wiel M. A., Terhaar Sive Droste J. S., Mongera S., Postma C., Meijerink W. J., Carvalho B., Meijer G. A. (2009). MiR-17-92 cluster is associated with 13q gain and c-myc expression during colorectal adenoma to adenocarcinoma progression. Br J Cancer.

[101] Guo S. T., Jiang C. C., Wang G. P., Li Y. P., Wang C. Y., Guo X. Y., Yang R. H., Feng Y., Wang F. H., Tseng H. Y., Thorne R. F., Jin L., Zhang X. D. (2013). MicroRNA-497 targets insulin-like growth factor 1 receptor and has a tumour suppressive role in human colorectal cancer. Oncogene.

[102] Brueckner B., Stresemann C., Kuner R., Mund C., Musch T., Meister M., Sultmann H., Lyko F. (2007). The human let-7a-3 locus contains an epigenetically regulated microRNA gene with oncogenic function. Cancer Res.

[103] Lujambio A., Ropero S., Ballestar E., Fraga M. F., Cerrato C., Setien F., Casado S., Suarez-Gauthier A., Sanchez-Cespedes M., Git A., Spiteri I., Das P. P., Caldas C. (2007). Genetic unmasking of an epigenetically silenced microRNA in human cancer cells. Cancer Res.

[104] Grady W. M., Parkin R. K., Mitchell P. S., Lee J. H., Kim Y. H., Tsuchiya K. D., Washington M. K., Paraskeva C., Willson J. K., Kaz A. M., Kroh E. M., Allen A., Fritz B. R. (2008). Epigenetic silencing of the intronic microRNA hsa-miR-342 and its host gene EVL in colorectal cancer. Oncogene.

[105] Lodygin D., Tarasov V., Epanchintsev A., Berking C., Knyazeva T., Korner H., Knyazev P., Diebold J., Hermeking H. (2008). Inactivation of miR-34a by aberrant CpG methylation in multiple types of cancer. Cell Cycle.

[106] Ito M., Mitsuhashi K., Igarashi H., Nosho K., Naito T., Yoshii S., Takahashi H., Fujita M., Sukawa Y., Yamamoto E., Takahashi T., Adachi Y., Nojima M. (2014). MicroRNA-31 expression in relation to BRAF mutation, CpG island methylation, colorectal continuum in serrated lesions. Int J Cancer.

[107] Slattery M. L., Wolff E., Hoffman M. D., Pellatt D. F., Milash B., Wolff R. K. (2011). MicroRNAs and colon and rectal cancer: differential expression by tumor location and subtype. Genes Chromosomes Cancer.

[108] Chivukula R. R., Shi G., Acharya A., Mills E. W., Zeitels L. R., Anandam J. L., Abdelnaby A. A., Balch G. C., Mansour J. C., Yopp A. C., Maitra A., Mendell J. T. (2014). An essential mesenchymal function for miR-143/145 in intestinal epithelial regeneration. Cell.

[109] Hofacker I. L. (2007). How microRNAs choose their targets. Nat Genet.

[110] Jacobsen A., Silber J., Harinath G., Huse J. T., Schultz N., Sander C. (2013). Analysis of microRNA-target interactions across diverse cancer types. Nat Struct Mol Biol.

[111] Clevers H., Loh K.M., Nusse R. (2014). Stem cell signaling. An integral program for tissue renewal and regeneration: Wnt signaling and stem cell control. Science.

[112] Attard T. M., Cuffari C., Tajouri T., Stoner J. A., Eisenberg M. T., Yardley J. H., Abraham S. C., Perry D., Vanderhoof J., Lynch H. (2004). Multicenter experience with upper gastrointestinal polyps in pediatric patients with familial adenomatous polyposis. Am J Gastroenterol.

[113] Miyoshi Y., Nagase H., Ando H., Horii A., Ichii S., Nakatsuru S., Aoki T., Miki Y., Mori T., Nakamura Y. (1992). Somatic mutations of the APC gene in colorectal tumors: mutation cluster region in the APC gene. Hum Mol Genet.

[114] Nagel R., Le Sage C., Diosdado B., Van Der Waal M., Oude Vrielink J. A., Bolijn A., Meijer G. A., Agami R (2008). Regulation of the adenomatous polyposis coli gene by the miR-135 family in colorectal cancer. Cancer Res.

[115] Valeri N., Braconi C., Gasparini P., Murgia C., Lampis A., Paulus-Hock V., Hart J. R., Ueno L., Grivennikov S. I., Lovat F., Paone A., Cascione L., Sumani K. M. (2014). MicroRNA-135b promotes cancer progression by acting as a downstream effector of oncogenic pathways in colon cancer. Cancer Cell.

[116] Clevers H (2006). Wnt/beta-catenin signaling in development and disease. Cell.

[117] Kaller M., Liffers S. T., Oeljeklaus S., Kuhlmann K., Roh S., Hoffmann R., Warscheid B., Hermeking H (2011). Genome-wide characterization of miR-34a induced changes in protein and mRNA expression by a combined pulsed SILAC and microarray analysis. Mol Cell Proteomics.

[118] Yamada N., Noguchi S., Mori T., Naoe T., Maruo K., Akao Y. (2013). Tumor-suppressive microRNA-145 targets catenin delta-1 to regulate Wnt/beta-catenin signaling in human colon cancer cells. Cancer Lett.

[119] Drebber U., Lay M., Wedemeyer I., Vallbohmer D., Bollschweiler E., Brabender J., Monig S. P., Holscher A. H., Dienes H. P., Odenthal M. (2011). Altered levels of the onco-microRNA 21 and the tumor-supressor microRNAs 143 and 145 in advanced rectal cancer indicate successful neoadjuvant chemoradiotherapy. Int J Oncol.

[120] Pichler M., Winter E., Stotz M., Eberhard K., Samonigg H., Lax S., Hoefler G. (2012). Down-regulation of KRAS-interacting miRNA-143 predicts poor prognosis but not response to EGFR-targeted agents in colorectal cancer. Br J Cancer.

[121] Zhang J., Zhang K., Bi M., Jiao X., Zhang D., Dong Q. (2014). Circulating microRNA expressions in colorectal cancer as predictors of response to chemotherapy. Anticancer Drugs.

[122] Schepeler T., Holm A., Halvey P., Nordentoft I., Lamy P., Riising E. M., Christensen L. L., Thorsen K., Liebler D. C., Helin K., Orntoft T. F., Andersen C. L. (2012). Attenuation of the beta-catenin/TCF4 complex in colorectal cancer cells induces several growth-suppressive microRNAs that target cancer promoting genes. Oncogene.

[123] Lan F., Yue X., Han L., Shi Z., Yang Y., Pu P., Yao Z., Kang C. (2012). Genome-wide identification of TCF7L2/TCF4 target miRNAs reveals a role for miR-21 in Wnt-driven epithelial cancer. Int J Oncol.

[124] Kim N. H., Cha Y. H., Kang S. E., Lee Y., Lee I., Cha S. Y., Ryu J. K., Na J. M., Park C., Yoon H. G., Park G. J., Yook J. I., Kim H. S. (2013). p53 regulates nuclear GSK-3 levels through miR-34-mediated Axin2 suppression in colorectal cancer cells. Cell Cycle.

[125] Kim N. H., Kim H. S., Kim N. G., Lee I., Choi H. S., Li X. Y., Kang S. E., Cha S. Y., Ryu J. K., Na J. M., Park C., Kim K., Lee S. (2011). p53 and microRNA-34 are suppressors of canonical Wnt signaling. Sci Signal.

[126] Takahashi M., Sung B., Shen Y., Hur K., Link A., Boland C. R., Aggarwal B. B., Goel A (2012). Boswellic acid exerts antitumor effects in colorectal cancer cells by modulating expression of the let-7 and miR-200 microRNA family. Carcinogenesis.

[127] Dews M., Fox J. L., Hultine S., Sundaram P., Wang W., Liu Y. Y., Furth E., Enders G. H., El-Deiry W., Schelter J. M., Cleary M. A., Thomas-Tikhonenko A (2010). The myc-miR-17∼92 axis blunts TGF{beta} signaling and production of multiple TGF{beta}-dependent antiangiogenic factors. Cancer Res.

[128] Oda K., Matsuoka Y., Funahashi A., Kitano H. (2005). A comprehensive pathway map of epidermal growth factor receptor signaling. Mol Syst Biol.

[129] Brink M., De Goeij A. F., Weijenberg M. P., Roemen G. M., Lentjes M. H., Pachen M. M., Smits K. M., De Bruine A. P., Goldbohm R. A., Van Den Brandt P. A. (2003). K-ras oncogene mutations in sporadic colorectal cancer in The Netherlands Cohort Study. Carcinogenesis.

[130] Saltz L. B., Meropol N. J., Loehrer P. J., Needle M. N., Kopit J., Mayer R. J. (2004). Phase II trial of cetuximab in patients with refractory colorectal cancer that expresses the epidermal growth factor receptor. J Clin Oncol.

[131] Dienstmann R., Salazar R., Tabernero J. (2015). Overcoming Resistance to Anti-EGFR Therapy in Colorectal Cancer. Am Soc Clin Oncol Educ Book.

[132] Johnson S. M., Grosshans H., Shingara J., Byrom M., Jarvis R., Cheng A., Labourier E., Reinert K. L., Brown D., Slack F. J. (2005). RAS is regulated by the let-7 microRNA family. Cell.

[133] Smits K. M., Paranjape T., Nallur S., Wouters K. A., Weijenberg M. P., Schouten L. J., Van Den Brandt P. A., Bosman F. T., Weidhaas J. B., Van Engeland M (2011). A let-7 microRNA SNP in the KRAS 3′UTR is prognostic in early-stage colorectal cancer. Clin Cancer Res.

[134] Ryan B. M., Robles A. I., Harris C. C. (2012). KRAS-LCS6 genotype as a prognostic marker in early-stage CRC--letter. Clin Cancer Res.

[135] Graziano F., Canestrari E., Loupakis F., Ruzzo A., Galluccio N., Santini D., Rocchi M., Vincenzi B., Salvatore L., Cremolini C., Spoto C., Catalano V., D'emidio S. (2010). Genetic modulation of the Let-7 microRNA binding to KRAS 3′-untranslated region and survival of metastatic colorectal cancer patients treated with salvage cetuximab-irinotecan. Pharmacogenomics J.

[136] Sha D., Lee A. M., Shi Q., Alberts S. R., Sargent D. J., Sinicrope F. A., Diasio R. B. (2014). Association study of the let-7 miRNA-complementary site variant in the 3′ untranslated region of the KRAS gene in stage III colon cancer (NCCTG N0147 Clinical Trial). Clin Cancer Res.

[137] Saridaki Z., Weidhaas J. B., Lenz H. J., Laurent-Puig P., Jacobs B., De Schutter J., De Roock W., Salzman D. W., Zhang W., Yang D., Pilati C., Bouche O., Piessevaux H. (2014). A let-7 microRNA-binding site polymorphism in KRAS predicts improved outcome in patients with metastatic colorectal cancer treated with salvage cetuximab/panitumumab monotherapy. Clin Cancer Res.

[138] Chen X., Guo X., Zhang H., Xiang Y., Chen J., Yin Y., Cai X., Wang K., Wang G., Ba Y., Zhu L., Wang J., Yang R. (2009). Role of miR-143 targeting KRAS in colorectal tumorigenesis. Oncogene.

[139] Tsang W. P., Kwok T.T. (2009). The miR-18a* microRNA functions as a potential tumor suppressor by targeting on K-Ras. Carcinogenesis.

[140] Hiraki M., Nishimura J., Takahashi H., Wu X., Takahashi Y., Miyo M., Nishida N., Uemura M., Hata T., Takemasa I., Mizushima T., Soh J. W., Doki Y. (2015). Concurrent Targeting of KRAS and AKT by MiR-4689 Is a Novel Treatment Against Mutant KRAS Colorectal Cancer. Mol Ther Nucleic Acids.

[141] Dong Y., Zhao J., Wu C. W., Zhang L., Liu X., Kang W., Leung W. W., Zhang N., Chan F. K., Sung J. J., Ng S. S., Yu J (2013). Tumor suppressor functions of miR-133a in colorectal cancer. Mol Cancer Res.

[142] Danielsen S. A., Eide P. W., Nesbakken A., Guren T., Leithe E., Lothe R. A. (2015). Portrait of the PI3K/AKT pathway in colorectal cancer. Biochim Biophys Acta.

[143] Arcaroli J. J., Quackenbush K. S., Powell R. W., Pitts T. M., Spreafico A., Varella-Garcia M., Bemis L., Tan A. C., Reinemann J. M., Touban B. M., Dasari A., Eckhardt S. G., Messersmith W. A. (2012). Common PIK3CA mutants and a novel 3′ UTR mutation are associated with increased sensitivity to saracatinib. Clin Cancer Res.

[144] Reddy S. M., Kopetz S., Morris J., Parikh N., Qiao W., Overman M. J., Fogelman D., Shureiqi I., Jacobs C., Malik Z., Jimenez C. A., Wolff R. A., Abbruzzese J. L. (2015). Phase II study of saracatinib (AZD0530) in patients with previously treated metastatic colorectal cancer. Invest New Drugs.

[145] Guo C., Sah J. F., Beard L., Willson J. K., Markowitz S. D., Guda K (2008). The noncoding RNA, miR-126, suppresses the growth of neoplastic cells by targeting phosphatidylinositol 3-kinase signaling and is frequently lost in colon cancers. Genes Chromosomes Cancer.

[146] Zhong M., Bian Z., Wu Z. (2013). miR-30a suppresses cell migration and invasion through downregulation of PIK3CD in colorectal carcinoma. Cell Physiol Biochem.

[147] Bonneau D., Longy M. (2000). Mutations of the human PTEN gene. Hum Mutat.

[148] Goel A., Arnold C. N., Niedzwiecki D., Carethers J. M., Dowell J. M., Wasserman L., Compton C., Mayer R. J., Bertagnolli M. M., Boland C. R. (2004). Frequent inactivation of PTEN by promoter hypermethylation in microsatellite instability-high sporadic colorectal cancers. Cancer Res.

[149] Humphreys K. J., Cobiac L., Le Leu R. K., Van Der Hoek M. B., Michael M. Z. (2013). Histone deacetylase inhibition in colorectal cancer cells reveals competing roles for members of the oncogenic miR-17-92 cluster. Mol Carcinog.

[150] Iliopoulos D., Jaeger S. A., Hirsch H. A., Bulyk M. L., Struhl K (2010). STAT3 activation of miR-21 and miR-181b-1 via PTEN and CYLD are part of the epigenetic switch linking inflammation to cancer. Mol Cell.

[151] Wu W., Yang J., Feng X., Wang H., Ye S., Yang P., Tan W., Wei G., Zhou Y. (2013). MicroRNA-32 (miR-32) regulates phosphatase and tensin homologue (PTEN) expression and promotes growth, migration, and invasion in colorectal carcinoma cells. Mol Cancer.

[152] Ragusa M., Statello L., Maugeri M., Majorana A., Barbagallo D., Salito L., Sammito M., Santonocito M., Angelica R., Cavallaro A., Scalia M., Caltabiano R., Privitera G. (2012). Specific alterations of the microRNA transcriptome and global network structure in colorectal cancer after treatment with MAPK/ERK inhibitors. J Mol Med (Berl).

[153] Bieging K. T., Mello S. S., Attardi L. D. (2014). Unravelling mechanisms of p53-mediated tumour suppression. Nat Rev Cancer.

[154] Hollstein M., Sidransky D., Vogelstein B., Harris C. C (1991). p53 mutations in human cancers. Science.

[155] Xi Y., Shalgi R., Fodstad O., Pilpel Y., Ju J. (2006). Differentially regulated micro-RNAs and actively translated messenger RNA transcripts by tumor suppressor p53 in colon cancer. Clin Cancer Res.

[156] He L., He X., Lim L. P., De Stanchina E., Xuan Z., Liang Y., Xue W., Zender L., Magnus J., Ridzon D., Jackson A. L., Linsley P. S., Chen C. (2007). A microRNA component of the p53 tumour suppressor network. Nature.

[157] Tarasov V., Jung P., Verdoodt B., Lodygin D., Epanchintsev A., Menssen A., Meister G., Hermeking H. (2007). Differential regulation of microRNAs by p53 revealed by massively parallel sequencing: miR-34a is a p53 target that induces apoptosis and G1-arrest. Cell Cycle.

[158] Toyota M., Suzuki H., Sasaki Y., Maruyama R., Imai K., Shinomura Y., Tokino T. (2008). Epigenetic silencing of microRNA-34b/c and B-cell translocation gene 4 is associated with CpG island methylation in colorectal cancer. Cancer Res.

[159] Akao Y., Noguchi S., Iio A., Kojima K., Takagi T., Naoe T. (2011). Dysregulation of microRNA-34a expression causes drug-resistance to 5-FU in human colon cancer DLD-1 cells. Cancer Lett.

[160] Yamakuchi M., Ferlito M., Lowenstein C. J. (2008). miR-34a repression of SIRT1 regulates apoptosis. Proc Natl Acad Sci U S A.

[161] Braun C. J., Zhang X., Savelyeva I., Wolff S., Moll U. M., Schepeler T., Orntoft T. F., Andersen C. L., Dobbelstein M (2008). p53-Responsive micrornas 192 and 215 are capable of inducing cell cycle arrest. Cancer Res.

[162] Nishida N., Yokobori T., Mimori K., Sudo T., Tanaka F., Shibata K., Ishii H., Doki Y., Kuwano H., Mori M. (2011). MicroRNA miR-125b is a prognostic marker in human colorectal cancer. Int J Oncol.

[163] Zhang C., Liu J., Wang X., Wu R., Lin M., Laddha S. V., Yang Q., Chan C. S., Feng Z (2014). MicroRNA-339-5p inhibits colorectal tumorigenesis through regulation of the MDM2/p53 signaling. Oncotarget.

[164] Jones M., Lal A. (2012). MicroRNAs, wild-type and mutant p53: more questions than answers. RNA Biol.

[165] Moustakas A., Heldin C.H. (2009). The regulation of TGFbeta signal transduction. Development.

[166] Biswas S., Trobridge P., Romero-Gallo J., Billheimer D., Myeroff L. L., Willson J. K., Markowitz S. D., Grady W. M. (2008). Mutational inactivation of TGFBR2 in microsatellite unstable colon cancer arises from the cooperation of genomic instability and the clonal outgrowth of transforming growth factor beta resistant cells. Genes Chromosomes Cancer.

[167] Bellam N., Pasche B. (2010). Tgf-beta signaling alterations and colon cancer. Cancer Treat Res.

[168] Yu Y., Kanwar S. S., Patel B. B., Oh P. S., Nautiyal J., Sarkar F. H., Majumdar A. P. (2012). MicroRNA-21 induces stemness by downregulating transforming growth factor beta receptor 2 (TGFbetaR2) in colon cancer cells. Carcinogenesis.

[169] Feng B., Dong T. T., Wang L. L., Zhou H. M., Zhao H. C., Dong F., Zheng M. H. (2012). Colorectal cancer migration and invasion initiated by microRNA-106a. PLoS One.

[170] Liu L., Nie J., Chen L., Dong G., Du X., Wu X., Tang Y., Han W. (2013). The oncogenic role of microRNA-130a/301a/454 in human colorectal cancer via targeting Smad4 expression. PLoS One.

[171] Zhang J., Lu Y., Yue X., Li H., Luo X., Wang Y., Wang K., Wan J. (2013). MiR-124 suppresses growth of human colorectal cancer by inhibiting STAT3. PLoS One.

[172] Liu M., Bi F., Zhou X., Zheng Y. (2012). Rho GTPase regulation by miRNAs and covalent modifications. Trends Cell Biol.

[173] Liu M., Lang N., Chen X., Tang Q., Liu S., Huang J., Zheng Y., Bi F. (2011). miR-185 targets RhoA and Cdc42 expression and inhibits the proliferation potential of human colorectal cells. Cancer Lett.

[174] Liu M., Lang N., Qiu M., Xu F., Li Q., Tang Q., Chen J., Chen X., Zhang S., Liu Z., Zhou J., Zhu Y., Deng Y. (2011). miR-137 targets Cdc42 expression, induces cell cycle G1 arrest and inhibits invasion in colorectal cancer cells. Int J Cancer.

[175] Hansen T. F., Andersen C. L., Nielsen B. S., Spindler K. L., Sorensen F. B., Lindebjerg J., Brandslund I., Jakobsen A (2011). Elevated microRNA-126 is associated with high vascular endothelial growth factor receptor 2 expression levels and high microvessel density in colorectal cancer. Oncol Lett.

[176] Ruzzo A., Graziano F., Vincenzi B., Canestrari E., Perrone G., Galluccio N., Catalano V., Loupakis F., Rabitti C., Santini D., Tonini G., Fiorentini G., Rossi D. (2012). High let-7a microRNA levels in KRAS-mutated colorectal carcinomas may rescue anti-EGFR therapy effects in patients with chemotherapy-refractory metastatic disease. Oncologist.

[177] Igarashi H., Kurihara H., Mitsuhashi K., Ito M., Okuda H., Kanno S., Naito T., Yoshii S., Takahashi H., Kusumi T., Hasegawa T., Sukawa Y., Adachi Y. (2014). Association of MicroRNA-31-5p with Clinical Efficacy of Anti-EGFR Therapy in Patients with Metastatic Colorectal Cancer. Ann Surg Oncol.

[178] Svoboda M., Izakovicova Holla L., Sefr R., Vrtkova I., Kocakova I., Tichy B., Dvorak J. (2008). Micro-RNAs miR125b and miR137 are frequently upregulated in response to capecitabine chemoradiotherapy of rectal cancer. Int J Oncol.

[179] Salendo J., Spitzner M., Kramer F., Zhang X., Jo P., Wolff H. A., Kitz J., Kaulfuss S., Beissbarth T., Dobbelstein M., Ghadimi M., Grade M., Gaedcke J. (2013). Identification of a microRNA expression signature for chemoradiosensitivity of colorectal cancer cells, involving miRNAs-320a, -224, -132 and let7g. Radiother Oncol.

[180] Kheirelseid E. A., Miller N., Chang K. H., Curran C., Hennessey E., Sheehan M., Newell J., Lemetre C., Balls G., Kerin M. J. (2013). miRNA expressions in rectal cancer as predictors of response to neoadjuvant chemoradiation therapy. Int J Colorectal Dis.

[181] Fang L., Li H., Wang L., Hu J., Jin T., Wang J., Yang B.B. (2014). MicroRNA-17-5p promotes chemotherapeutic drug resistance and tumour metastasis of colorectal cancer by repressing PTEN expression. Oncotarget.

[182] Liu K., Li G., Fan C., Zhou X., Wu B., Li J. (2011). Increased expression of microRNA-21and its association with chemotherapeutic response in human colorectal cancer. J Int Med Res.

[183] Oue N., Anami K., Schetter A. J., Moehler M., Okayama H., Khan M. A., Bowman E. D., Mueller A., Schad A., Shimomura M., Hinoi T., Aoyagi K., Sasaki H. (2014). High miR-21 expression from FFPE tissues is associated with poor survival and response to adjuvant chemotherapy in colon cancer. Int J Cancer.

[184] Molina-Pinelo S., Carnero A., Rivera F., Estevez-Garcia P., Bozada J. M., Limon M. L., Benavent M., Gomez J., Pastor M. D., Chaves M., Suarez R., Paz-Ares L., De La Portilla F. (2014). MiR-107 and miR-99a-3p predict chemotherapy response in patients with advanced colorectal cancer. BMC Cancer.

[185] Della Vittoria Scarpati G., Falcetta F., Carlomagno C., Ubezio P., Marchini S., De Stefano A., Singh V. K., D'incalci M., De Placido S., Pepe S. (2012). A specific miRNA signature correlates with complete pathological response to neoadjuvant chemoradiotherapy in locally advanced rectal cancer. Int J Radiat Oncol Biol Phys.

[186] Hansen T. F., Nielsen B. S., Sorensen F. B., Johnsson A., Jakobsen A (2014). Epidermal growth factor-like domain 7 predicts response to first-line chemotherapy and bevacizumab in patients with metastatic colorectal cancer. Mol Cancer Ther.

[187] Qian X., Yu J., Yin Y., He J., Wang L., Li Q., Zhang L. Q., Li C. Y., Shi Z. M., Xu Q., Li W., Lai L. H., Liu L. Z. (2013). MicroRNA-143 inhibits tumor growth and angiogenesis and sensitizes chemosensitivity to oxaliplatin in colorectal cancers. Cell Cycle.

[188] Takahashi M., Cuatrecasas M., Balaguer F., Hur K., Toiyama Y., Castells A., Boland C.R., Goel A. (2012). The clinical significance of MiR-148a as a predictive biomarker in patients with advanced colorectal cancer. PLoS One.

[189] Song Y., Xu Y., Wang Z., Chen Y., Yue Z., Gao P., Xing C., Xu H. (2012). MicroRNA-148b suppresses cell growth by targeting cholecystokinin-2 receptor in colorectal cancer. Int J Cancer.

[190] Dou R., Deng Y., Huang L., Fu S., Tan S., Wang L., Lian L., Fang L., Fan X., Jin G., Liu H., Wang J. (2013). Multi-microarray identifies lower AQP9 expression in adjuvant chemotherapy nonresponders with stage III colorectal cancer. Cancer Lett.

[191] Bhangu A., Wood G., Brown G., Darzi A., Tekkis P., Goldin R. (2014). The role of epithelial mesenchymal transition and resistance to neoadjuvant therapy in locally advanced rectal cancer. Colorectal Dis.

[192] Chen D. L., Wang Z. Q., Zeng Z. L., Wu W. J., Zhang D. S., Luo H. Y., Wang F., Qiu M. Z., Wang D. S., Ren C., Wang F. H., Chiao L. J., Pelicano H. (2014). Identification of microRNA-214 as a negative regulator of colorectal cancer liver metastasis by way of regulation of fibroblast growth factor receptor 1 expression. Hepatology.

[193] Song B., Wang Y., Titmus M. A., Botchkina G., Formentini A., Kornmann M., Ju J (2010). Molecular mechanism of chemoresistance by miR-215 in osteosarcoma and colon cancer cells. Mol Cancer.

[194] Svoboda M., Sana J., Fabian P., Kocakova I., Gombosova J., Nekvindova J., Radova L., Vyzula R., Slaby O. (2012). MicroRNA expression profile associated with response to neoadjuvant chemoradiotherapy in locally advanced rectal cancer patients. Radiat Oncol.

[195] Wan L. Y., Deng J., Xiang X. J., Zhang L., Yu F., Chen J., Sun Z., Feng M., Xiong J. P. (2015). miR-320 enhances the sensitivity of human colon cancer cells to chemoradiotherapy in vitro by targeting FOXM1. Biochem Biophys Res Commun.

[196] Bitarte N., Bandres E., Boni V., Zarate R., Rodriguez J., Gonzalez-Huarriz M., Lopez I., Javier Sola J., Alonso M. M., Fortes P., Garcia-Foncillas J (2011). MicroRNA-451 is involved in the self-renewal, tumorigenicity, and chemoresistance of colorectal cancer stem cells. Stem Cells.

[197] Rasmussen M. H., Jensen N. F., Tarpgaard L. S., Qvortrup C., Romer M. U., Stenvang J., Hansen T. P., Christensen L. L., Lindebjerg J., Hansen F., Jensen B. V., Hansen T. F., Pfeiffer P. (2013). High expression of microRNA-625-3p is associated with poor response to first-line oxaliplatin based treatment of metastatic colorectal cancer. Mol Oncol.

[198] Fassan M., Pizzi M., Giacomelli L., Mescoli C., Ludwig K., Pucciarelli S., Rugge M. (2011). PDCD4 nuclear loss inversely correlates with miR-21 levels in colon carcinogenesis. Virchows Arch.

[199] Yamamichi N., Shimomura R., Inada K., Sakurai K., Haraguchi T., Ozaki Y., Fujita S., Mizutani T., Furukawa C., Fujishiro M., Ichinose M., Shiogama K., Tsutsumi Y. (2009). Locked nucleic acid in situ hybridization analysis of miR-21 expression during colorectal cancer development. Clin Cancer Res.

[200] Mori Y., Olaru A. V., Cheng Y., Agarwal R., Yang J., Luvsanjav D., Yu W., Selaru F. M., Hutfless S., Lazarev M., Kwon J. H., Brant S. R., Marohn M. R. (2011). Novel candidate colorectal cancer biomarkers identified by methylation microarray-based scanning. Endocr Relat Cancer.

[201] Yang Y., Peng W., Tang T., Xia L., Wang X. D., Duan B. F., Shu Y (2014). MicroRNAs as promising biomarkers for tumor-staging: evaluation of MiR21 MiR155 MiR29a and MiR92a in predicting tumor stage of rectal cancer. Asian Pac J Cancer Prev.

[202] Wang Z. H., Ren L. L., Zheng P., Zheng H. M., Yu Y. N., Wang J. L., Lin Y. W., Chen Y. X., Ge Z. Z., Chen X. Y., Hong J., Fang J. Y. (2014). miR-194 as a predictor for adenoma recurrence in patients with advanced colorectal adenoma after polypectomy. Cancer Prev Res (Phila).

[203] Peng J., Xie Z., Cheng L., Zhang Y., Chen J., Yu H., Li Z., Kang H. (2015). Paired design study by real-time PCR: miR-378* and miR-145 are potent early diagnostic biomarkers of human colorectal cancer. BMC Cancer.

[204] Hansen T. F., Kjaer-Frifeldt S., Christensen R. D., Morgenthaler S., Blondal T., Lindebjerg J., Sorensen F. B., Jakobsen A (2014). Redefining high-risk patients with stage II colon cancer by risk index and microRNA-21: results from a population-based cohort. Br J Cancer.

[205] Schetter A. J., Leung S. Y., Sohn J. J., Zanetti K. A., Bowman E. D., Yanaihara N., Yuen S. T., Chan T. L., Kwong D. L., Au G. K., Liu C. G., Calin G. A., Croce C. M. (2008). MicroRNA expression profiles associated with prognosis and therapeutic outcome in colon adenocarcinoma. JAMA.

[206] Schetter A. J., Nguyen G. H., Bowman E. D., Mathe E. A., Yuen S. T., Hawkes J. E., Croce C. M., Leung S. Y., Harris C. C. (2009). Association of inflammation-related and microRNA gene expression with cancer-specific mortality of colon adenocarcinoma. Clin Cancer Res.

[207] Xia X., Yang B., Zhai X., Liu X., Shen K., Wu Z., Cai J. (2013). Prognostic role of microRNA-21 in colorectal cancer: a meta-analysis. PLoS One.

[208] Zhang H., Li P., Ju H., Pesta M., Kulda V., Jin W., Cai M., Liu C., Wu H., Xu J., Ye Y., Zhang G., Xu E. (2014). Diagnostic and prognostic value of microRNA-21 in colorectal cancer: an original study and individual participant data meta-analysis. Cancer Epidemiol Biomarkers Prev.

[209] Shibuya H., Iinuma H., Shimada R., Horiuchi A., Watanabe T. (2010). Clinicopathological and prognostic value of microRNA-21 and microRNA-155 in colorectal cancer. Oncology.

[210] Wang F., Zhang P., Ma Y., Yang J., Moyer M. P., Shi C., Peng J., Qin H (2012). NIRF is frequently upregulated in colorectal cancer and its oncogenicity can be suppressed by let-7a microRNA. Cancer Lett.

[211] Han H. B., Gu J., Zuo H. J., Chen Z. G., Zhao W., Li M., Ji D. B., Lu Y. Y., Zhang Z. Q. (2012). Let-7c functions as a metastasis suppressor by targeting MMP11 and PBX3 in colorectal cancer. J Pathol.

[212] Rohr C., Kerick M., Fischer A., Kuhn A., Kashofer K., Timmermann B., Daskalaki A., Meinel T., Drichel D., Borno S. T., Nowka A., Krobitsch S., Mchardy A. C. (2013). High-throughput miRNA and mRNA sequencing of paired colorectal normal, tumor and metastasis tissues, bioinformatic modeling of miRNA-1 therapeutic applications. PLoS One.

[213] Strillacci A., Valerii M. C., Sansone P., Caggiano C., Sgromo A., Vittori L., Fiorentino M., Poggioli G., Rizzello F., Campieri M., Spisni E (2013). Loss of miR-101 expression promotes Wnt/beta-catenin signalling pathway activation and malignancy in colon cancer cells. J Pathol.

[214] Ak S., Tunca B., Tezcan G., Cecener G., Egeli U., Yilmazlar T., Ozturk E., Yerci O. (2014). MicroRNA expression patterns of tumors in early-onset colorectal cancer patients. J Surg Res.

[215] Hansen T. F., Kjaer-Frifeldt S., Morgenthaler S., Blondal T., Lindebjerg J., Jakobsen A., Sorensen F. B. (2014). The prognostic value of microRNA-126 and microvessel density in patients with stage II colon cancer: results from a population cohort. J Transl Med.

[216] Colangelo T., Fucci A., Votino C., Sabatino L., Pancione M., Laudanna C., Binaschi M., Bigioni M., Maggi C. A., Parente D., Forte N., Colantuoni V (2013). MicroRNA-130b promotes tumor development and is associated with poor prognosis in colorectal cancer. Neoplasia.

[217] Liu X., Duan B., Dong Y., He C., Zhou H., Sheng H., Gao H., Zhang X. (2014). MicroRNA-139-3p indicates a poor prognosis of colon cancer. Int J Clin Exp Pathol.

[218] Chang K. H., Miller N., Kheirelseid E. A., Lemetre C., Ball G. R., Smith M. J., Regan M., Mcanena O. J., Kerin M. J. (2011). MicroRNA signature analysis in colorectal cancer: identification of expression profiles in stage II tumors associated with aggressive disease. Int J Colorectal Dis.

[219] Chen Y., Song Y., Wang Z., Yue Z., Xu H., Xing C., Liu Z. (2010). Altered expression of MiR-148a and MiR-152 in gastrointestinal cancers and its clinical significance. J Gastrointest Surg.

[220] Xiao G., Tang H., Wei W., Li J., Ji L., Ge J. (2014). Aberrant Expression of MicroRNA-15a and MicroRNA-16 Synergistically Associates with Tumor Progression and Prognosis in Patients with Colorectal Cancer. Gastroenterol Res Pract.

[221] Aslam M. I., Venkatesh J., Jameson J. S., West K., Pringle J. H., Singh B. (2014). MicroRNA expression profiling based identification of high risk Dukes' Stage B colorectal cancer: a preliminary study. Colorectal Dis.

[222] Yu G., Tang J. Q., Tian M. L., Li H., Wang X., Wu T., Zhu J., Huang S. J., Wan Y. L. (2012). Prognostic values of the miR-17-92 cluster and its paralogs in colon cancer. J Surg Oncol.

[223] Zhou T., Zhang G. J., Zhou H., Xiao H. X., Li Y. (2014). Overexpression of microRNA-183 in human colorectal cancer and its clinical significance. Eur J Gastroenterol Hepatol.

[224] Wang X., Wang J., Ma H., Zhang J., Zhou X. (2012). Downregulation of miR-195 correlates with lymph node metastasis and poor prognosis in colorectal cancer. Med Oncol.

[225] Piepoli A., Tavano F., Copetti M., Mazza T., Palumbo O., Panza A., Di Mola F. F., Pazienza V., Mazzoccoli G., Biscaglia G., Gentile A., Mastrodonato N., Carella M. (2012). Mirna expression profiles identify drivers in colorectal and pancreatic cancers. PLoS One.

[226] Ge J., Chen Z., Li R., Lu T., Xiao G. (2014). Upregulation of microRNA-196a and microRNA-196b cooperatively correlate with aggressive progression and unfavorable prognosis in patients with colorectal cancer. Cancer Cell Int.

[227] Mariani M., He S., Mchugh M., Andreoli M., Pandya D., Sieber S., Wu Z., Fiedler P., Shahabi S., Ferlini C. (2014). Integrated multidimensional analysis is required for accurate prognostic biomarkers in colorectal cancer. PLoS One.

[228] Karaayvaz M., Pal T., Song B., Zhang C., Georgakopoulos P., Mehmood S., Burke S., Shroyer K., Ju J. (2011). Prognostic significance of miR-215 in colon cancer. Clin Colorectal Cancer.

[229] Liao W. T., Li T. T., Wang Z. G., Wang S. Y., He M. R., Ye Y. P., Qi L., Cui Y. M., Wu P., Jiao H. L., Zhang C., Xie Y. J., Wang J. X. (2013). microRNA-224 promotes cell proliferation and tumor growth in human colorectal cancer by repressing PHLPP1 and PHLPP2. Clin Cancer Res.

[230] Sun K., Su G., Deng H., Dong J., Lei S., Li G. (2014). Relationship between miRNA-338-3p expression and progression and prognosis of human colorectal carcinoma. Chin Med J (Engl).

[231] Takeyama H., Yamamoto H., Yamashita S., Wu X., Takahashi H., Nishimura J., Haraguchi N., Miyake Y., Suzuki R., Murata K., Ohue M., Kato T., Takemasa I. (2014). Decreased miR-340 expression in bone marrow is associated with liver metastasis of colorectal cancer. Mol Cancer Ther.

[232] Dai X., Chiang Y., Wang Z., Song Y., Lu C., Gao P., Xu H. (2012). Expression levels of microRNA-375 in colorectal carcinoma. Mol Med Rep.

[233] Li J., Du L., Yang Y., Wang C., Liu H., Wang L., Zhang X., Li W., Zheng G., Dong Z. (2013). MiR-429 is an independent prognostic factor in colorectal cancer and exerts its anti-apoptotic function by targeting SOX2. Cancer Lett.

[234] Bullock M. D., Pickard K. M., Nielsen B. S., Sayan A. E., Jenei V., Mellone M., Mitter R., Primrose J. N., Thomas G. J., Packham G. K., Mirnezami A. H. (2013). Pleiotropic actions of miR-21 highlight the critical role of deregulated stromal microRNAs during colorectal cancer progression. Cell Death Dis.

[235] Drusco A., Nuovo G. J., Zanesi N., Di Leva G., Pichiorri F., Volinia S., Fernandez C., Antenucci A., Costinean S., Bottoni A., Rosito I. A., Liu C. G., Burch A. (2014). MicroRNA profiles discriminate among colon cancer metastasis. PLoS One.

[236] Goossens-Beumer I. J., Derr R. S., Buermans H. P., Goeman J. J., Bohringer S., Morreau H., Nitsche U., Janssen K. P., Van De Velde C. J., Kuppen P. J. (2015). MicroRNA classifier and nomogram for metastasis prediction in colon cancer. Cancer Epidemiol Biomarkers Prev.

[237] Bao Y., Chen Z., Guo Y., Feng Y., Li Z., Han W., Wang J., Zhao W., Jiao Y., Li K., Wang Q., Wang J., Zhang H. (2014). Tumor suppressor microRNA-27a in colorectal carcinogenesis and progression by targeting SGPP1 and Smad2. PLoS One.

[238] Iino I., Kikuchi H., Miyazaki S., Hiramatsu Y., Ohta M., Kamiya K., Kusama Y., Baba S., Setou M., Konno H. (2013). Effect of miR-122 and its target gene cationic amino acid transporter 1 on colorectal liver metastasis. Cancer Sci.

[239] Wang L. L., Du L. T., Li J., Liu Y. M., Qu A. L., Yang Y. M., Zhang X., Zheng G. X., Wang C. X. (2014). Decreased expression of miR-133a correlates with poor prognosis in colorectal cancer patients. World J Gastroenterol.

[240] Duan F. T., Qian F., Fang K., Lin K. Y., Wang W. T., Chen Y. Q. (2013). miR-133b, a muscle-specific microRNA, is a novel prognostic marker that participates in the progression of human colorectal cancer via regulation of CXCR4 expression. Mol Cancer.

[241] Lin C. W., Li X. R., Zhang Y., Hu G., Guo Y. H., Zhou J. Y., Du J., Lv L., Gao K., Zhang Y., Deng H (2014). TAp63 suppress metastasis via miR-133b in colon cancer cells. Br J Cancer.

[242] Zhang Z., Liu X., Feng B., Liu N., Wu Q., Han Y., Nie Y., Wu K., Shi Y., Fan D. (2015). STIM1, a direct target of microRNA-185, promotes tumor metastasis, is associated with poor prognosis in colorectal cancer. Oncogene.

[243] Akcakaya P., Ekelund S., Kolosenko I., Caramuta S., Ozata D. M., Xie H., Lindforss U., Olivecrona H., Lui W. O. (2011). miR-185 and miR-133b deregulation is associated with overall survival and metastasis in colorectal cancer. Int J Oncol.

[244] Zhang P., Ji D. B., Han H. B., Shi Y. F., Du C. Z., Gu J (2014). Downregulation of miR-193a-5p correlates with lymph node metastasis and poor prognosis in colorectal cancer. World J Gastroenterol.

[245] Hu Y., Liu J., Jiang B., Chen J., Fu Z., Bai F., Jiang J., Tang Z. (2014). MiR-199a-5p loss up-regulated DDR1 aggravated colorectal cancer by activating epithelial-to-mesenchymal transition related signaling. Dig Dis Sci.

[246] Qu A., Du L., Yang Y., Liu H., Li J., Wang L., Liu Y., Dong Z., Zhang X., Jiang X., Wang H., Li Z., Zheng G. (2014). Hypoxia-inducible MiR-210 is an independent prognostic factor and contributes to metastasis in colorectal cancer. PLoS One.

[247] Ellermeier C., Vang S., Cleveland K., Durand W., Resnick M. B., Brodsky A. S. (2014). Prognostic microRNA expression signature from examination of colorectal primary and metastatic tumors. Anticancer Res.

[248] Ke T. W., Hsu H. L., Wu Y. H., Chen W. T., Cheng Y. W., Cheng C. W. (2014). MicroRNA-224 suppresses colorectal cancer cell migration by targeting Cdc42. Dis Markers.

[249] Yuan K., Xie K., Fox J., Zeng H., Gao H., Huang C., Wu M. (2013). Decreased levels of miR-224 and the passenger strand of miR-221 increase MBD2, suppressing maspin and promoting colorectal tumor growth, metastasis in mice. Gastroenterology.

[250] Lou X., Qi X., Zhang Y., Long H., Yang J. (2013). Decreased expression of microRNA-625 is associated with tumor metastasis and poor prognosis in patients with colorectal cancer. J Surg Oncol.

[251] Kang W. K., Lee J. K., Oh S. T., Lee S. H., Jung C. K. (2015). Stromal expression of miR-21 in T3-4a colorectal cancer is an independent predictor of early tumor relapse. BMC Gastroenterol.

[252] Li S., Gao J., Gu J., Yuan J., Hua D., Shen L. (2013). MicroRNA-215 inhibits relapse of colorectal cancer patients following radical surgery. Med Oncol.

[253] Hansen T. F., Christensen Rd, Andersen R. F., Sorensen F. B., Johnsson A., Jakobsen A. (2013). MicroRNA-126 and epidermal growth factor-like domain 7-an angiogenic couple of importance in metastatic colorectal cancer. Results from the Nordic ACT trial. Br J Cancer.

[254] Schou J. V., Rossi S., Jensen B. V., Nielsen D. L., Pfeiffer P., Hogdall E., Yilmaz M., Tejpar S., Delorenzi M., Kruhoffer M., Johansen J. S. (2014). miR-345 in metastatic colorectal cancer: a non-invasive biomarker for clinical outcome in non-KRAS mutant patients treated with 3rd line cetuximab and irinotecan. PLoS One.

[255] Chen Q., Xia H. W., Ge X. J., Zhang Y. C., Tang Q. L., Bi F. (2013). Serum miR-19a predicts resistance to FOLFOX chemotherapy in advanced colorectal cancer cases. Asian Pac J Cancer Prev.

[256] Hansen T. F., Carlsen A. L., Heegaard N. H., Sorensen F. B., Jakobsen A. (2015). Changes in circulating microRNA-126 during treatment with chemotherapy and bevacizumab predicts treatment response in patients with metastatic colorectal cancer. Br J Cancer.

[257] Chen J., Wang W., Zhang Y., Chen Y., Hu T. (2014). Predicting distant metastasis and chemoresistance using plasma miRNAs. Med Oncol.

[258] Faltejskova P., Bocanek O., Sachlova M., Svoboda M., Kiss I., Vyzula R., Slaby O. (2012). Circulating miR-17-3p, miR-29a, miR-92a and miR-135b in serum: Evidence against their usage as biomarkers in colorectal cancer. Cancer Biomark.

[259] Giraldez M. D., Lozano J. J., Ramirez G., Hijona E., Bujanda L., Castells A., Gironella M. (2013). Circulating microRNAs as biomarkers of colorectal cancer: results from a genome-wide profiling and validation study. Clin Gastroenterol Hepatol.

[260] Zheng G., Du L., Yang X., Zhang X., Wang L., Yang Y., Li J., Wang C. (2014). Serum microRNA panel as biomarkers for early diagnosis of colorectal adenocarcinoma. Br J Cancer.

[261] Toiyama Y., Takahashi M., Hur K., Nagasaka T., Tanaka K., Inoue Y., Kusunoki M., Boland C.R., Goel A. (2013). Serum miR-21 as a diagnostic and prognostic biomarker in colorectal cancer. J Natl Cancer Inst.

[262] Basati G., Emami Razavi A., Abdi S., Mirzaei A. (2014). Elevated level of microRNA-21 in the serum of patients with colorectal cancer. Med Oncol.

[263] Wang B., Zhang Q. (2012). The expression and clinical significance of circulating microRNA-21 in serum of five solid tumors. J Cancer Res Clin Oncol.

[264] Wang J., Huang S. K., Zhao M., Yang M., Zhong J. L., Gu Y. Y., Peng H., Che Y. Q., Huang C. Z. (2014). Identification of a circulating microRNA signature for colorectal cancer detection. PLoS One.

[265] Zanutto S., Pizzamiglio S., Ghilotti M., Bertan C., Ravagnani F., Perrone F., Leo E., Pilotti S., Verderio P., Gariboldi M., Pierotti M.A. (2014). Circulating miR-378 in plasma: a reliable, haemolysis-independent biomarker for colorectal cancer. Br J Cancer.

[266] Du M., Liu S., Gu D., Wang Q., Zhu L., Kang M., Shi D., Chu H., Tong N., Chen J., Adams T. S., Zhang Z., Wang M (2014). Clinical potential role of circulating microRNAs in early diagnosis of colorectal cancer patients. Carcinogenesis.

[267] Huang Z., Huang D., Ni S., Peng Z., Sheng W., Du X. (2010). Plasma microRNAs are promising novel biomarkers for early detection of colorectal cancer. Int J Cancer.

[268] Nugent M., Miller N., Kerin M.J. (2012). Circulating miR-34a levels are reduced in colorectal cancer. J Surg Oncol.

[269] Yin Y., Yan Z. P., Lu N. N., Xu Q., He J., Qian X., Yu J., Guan X., Jiang B. H., Liu L. Z. (2013). Downregulation of miR-145 associated with cancer progression and VEGF transcriptional activation by targeting N-RAS and IRS1. Biochim Biophys Acta.

[270] Ramzy I., Hasaballah M., Marzaban R., Shaker O., Soliman Z.A. (2015). Evaluation of microRNAs-29a, 92a and 145 in colorectal carcinoma as candidate diagnostic markers: An Egyptian pilot study. Clin Res Hepatol Gastroenterol.

[271] Yong F. L., Law C. W., Wang C. W. (2013). Potentiality of a triple microRNA classifier: miR-193a-3p, miR-23a and miR-338-5p for early detection of colorectal cancer. BMC Cancer.

[272] Xu L., Li M., Wang M., Yan D., Feng G., An G. (2014). The expression of microRNA-375 in plasma and tissue is matched in human colorectal cancer. BMC Cancer.

[273] Wang S., Xiang J., Li Z., Lu S., Hu J., Gao X., Yu L., Wang L., Wang J., Wu Y., Chen Z., Zhu H. (2015). A plasma microRNA panel for early detection of colorectal cancer. Int J Cancer.

[274] Hofsli E., Sjursen W., Prestvik W. S., Johansen J., Rye M., Trano G., Wasmuth H. H., Hatlevoll I., Thommesen L (2013). Identification of serum microRNA profiles in colon cancer. Br J Cancer.

[275] Lu X., Lu J. (2015). The significance of detection of serum miR-423-5p and miR-484 for diagnosis of colorectal cancer. Clin Lab.

[276] Kanaan Z., Roberts H., Eichenberger M. R., Billeter A., Ocheretner G., Pan J., Rai S. N., Jorden J., Williford A., Galandiuk S (2013). A plasma microRNA panel for detection of colorectal adenomas: a step toward more precise screening for colorectal cancer. Ann Surg.

[277] Wang Q., Huang Z., Ni S., Xiao X., Xu Q., Wang L., Huang D., Tan C., Sheng W., Du X. (2012). Plasma miR-601 and miR-760 are novel biomarkers for the early detection of colorectal cancer. PLoS One.

[278] Ahmed F. E., Amed N. C., Vos P. W., Bonnerup C., Atkins J. N., Casey M., Nuovo G. J., Naziri W., Wiley J. E., Allison R. R. (2012). Diagnostic microRNA markers to screen for sporadic human colon cancer in blood. Cancer Genomics Proteomics.

[279] Menendez P., Padilla D., Villarejo P., Palomino T., Nieto P., Menendez J. M., Rodriguez-Montes J. A. (2013). Prognostic implications of serum microRNA-21 in colorectal cancer. J Surg Oncol.

[280] Jinushi T., Shibayama Y., Kinoshita I., Oizumi S., Jinushi M., Aota T., Takahashi T., Horita S., Dosaka-Akita H., Iseki K. (2014). Low expression levels of microRNA-124-5p correlated with poor prognosis in colorectal cancer via targeting of SMC4. Cancer Med.

[281] Cheng H., Zhang L., Cogdell D. E., Zheng H., Schetter A. J., Nykter M., Harris C. C., Chen K., Hamilton S. R., Zhang W (2011). Circulating plasma MiR-141 is a novel biomarker for metastatic colon cancer and predicts poor prognosis. PLoS One.

[282] Lv Z. C., Fan Y. S., Chen H. B., Zhao D. W. (2015). Investigation of microRNA-155 as a serum diagnostic and prognostic biomarker for colorectal cancer. Tumour Biol.

[283] Toiyama Y., Hur K., Tanaka K., Inoue Y., Kusunoki M., Boland C.R., Goel A. (2014). Serum miR-200c is a novel prognostic and metastasis-predictive biomarker in patients with colorectal cancer. Ann Surg.

[284] Yu H., Gao G., Jiang L., Guo L., Lin M., Jiao X., Jia W., Huang J. (2013). Decreased expression of miR-218 is associated with poor prognosis in patients with colorectal cancer. Int J Clin Exp Pathol.

[285] Yong F. L., Wang C. W., Roslani A. C., Law C. W. (2014). The involvement of miR-23a/APAF1 regulation axis in colorectal cancer. Int J Mol Sci.

[286] Wang L.G., Gu J. (2012). Serum microRNA-29a is a promising novel marker for early detection of colorectal liver metastasis. Cancer Epidemiol.

[287] Yamada N., Nakagawa Y., Tsujimura N., Kumazaki M., Noguchi S., Mori T., Hirata I., Maruo K., Akao Y. (2013). Role of Intracellular and Extracellular MicroRNA-92a in Colorectal Cancer. Transl Oncol.

[288] Hur K., Toiyama Y., Schetter A. J., Okugawa Y., Harris C. C., Boland C. R., Goel A. (2015). Identification of a metastasis-specific MicroRNA signature in human colorectal cancer. J Natl Cancer Inst.

[289] Yin J., Bai Z., Song J., Yang Y., Wang J., Han W., Zhang J., Meng H., Ma X., Yang Y., Wang T., Li W., Zhang Z. (2014). Differential expression of serum miR-126, miR-141 and miR-21 as novel biomarkers for early detection of liver metastasis in colorectal cancer. Chin J Cancer Res.

[290] Shivapurkar N., Weiner L. M., Marshall J. L., Madhavan S., Deslattes Mays A., Juhl H., Wellstein A. (2014). Recurrence of early stage colon cancer predicted by expression pattern of circulating microRNAs. PLoS One.

[291] Brunet Vega A., Pericay C., Moya I., Ferrer A., Dotor E., Pisa A., Casalots A., Serra-Aracil X., Oliva J. C., Ruiz A., Saigi E (2013). microRNA expression profile in stage III colorectal cancer: circulating miR-18a and miR-29a as promising biomarkers. Oncol Rep.

[292] Yang I. P., Tsai H. L., Huang C. W., Huang M. Y., Hou M. F., Juo S. H., Wang J. Y. (2013). The functional significance of microRNA-29c in patients with colorectal cancer: a potential circulating biomarker for predicting early relapse. PLoS One.

[293] Tsai H. L., Yang I. P., Huang C. W., Ma C. J., Kuo C. H., Lu C. Y., Juo S. H., Wang J. Y. (2013). Clinical significance of microRNA-148a in patients with early relapse of stage II stage and III colorectal cancer after curative resection. Transl Res.

[294] Ahmed F. E., Ahmed N. C., Vos P. W., Bonnerup C., Atkins J. N., Casey M., Nuovo G. J., Naziri W., Wiley J. E., Mota H., Allison R. R. (2013). Diagnostic microRNA markers to screen for sporadic human colon cancer in stool: I. Proof of principle. Cancer Genomics Proteomics.

[295] Link A., Balaguer F., Shen Y., Nagasaka T., Lozano J. J., Boland C. R., Goel A. (2010). Fecal MicroRNAs as novel biomarkers for colon cancer screening. Cancer Epidemiol Biomarkers Prev.

[296] Wu X. D., Song Y. C., Cao P. L., Zhang H., Guo Q., Yan R., Diao D. M., Cheng Y., Dang C. X. (2014). Detection of miR-34a and miR-34b/c in stool sample as potential screening biomarkers for noninvasive diagnosis of colorectal cancer. Med Oncol.

[297] Kalimutho M., Di Cecilia S., Del Vecchio Blanco G., Roviello F., Sileri P., Cretella M., Formosa A., Corso G., Marrelli D., Pallone F., Federici G., Bernardini S. (2011). Epigenetically silenced miR-34b/c as a novel faecal-based screening marker for colorectal cancer. Br J Cancer.

[298] Wu C. W., Ng S. C., Dong Y., Tian L., Ng S. S., Leung W. W., Law W. T., Yau T. O., Chan F. K., Sung J. J., Yu J (2014). Identification of microRNA-135b in stool as a potential noninvasive biomarker for colorectal cancer and adenoma. Clin Cancer Res.

[299] Li J. M., Zhao R. H., Li S. T., Xie C. X., Jiang H. H., Ding W. J., Du P., Chen W., Yang M., Cui L. (2012). Down-regulation of fecal miR-143 and miR-145 as potential markers for colorectal cancer. Saudi Med J.

[300] Kalimutho M., Del Vecchio Blanco G., Di Cecilia S., Sileri P., Cretella M., Pallone F., Federici G., Bernardini S. (2011). Differential expression of miR-144* as a novel fecal-based diagnostic marker for colorectal cancer. J Gastroenterol.

[301] Yau T. O., Wu C. W., Dong Y., Tang C. M., Ng S. S., Chan F. K., Sung J. J., Yu J. (2014). microRNA-221 and microRNA-18a identification in stool as potential biomarkers for the non-invasive diagnosis of colorectal carcinoma. Br J Cancer.

[302] Phua L. C., Chue X. P., Koh P. K., Cheah P. Y., Chan E. C., Ho H. K. (2014). Global fecal microRNA profiling in the identification of biomarkers for colorectal cancer screening among Asians. Oncol Rep.

[303] Link A., Balaguer F., Nagasaka T., Boland C.R., Goel A. (2014). MicroRNA miR-J1-5p as a potential biomarker for JC virus infection in the gastrointestinal tract. PLoS One.

[304] Wu C. W., Ng S. S., Dong Y. J., Ng S. C., Leung W. W., Lee C. W., Wong Y. N., Chan F. K., Yu J., Sung J. J. (2012). Detection of miR-92a and miR-21 in stool samples as potential screening biomarkers for colorectal cancer and polyps. Gut.

[305] Rotelli M. T., Di Lena M., Cavallini A., Lippolis C., Bonfrate L., Chetta N., Portincasa P., Altomare D. F. (2015). Fecal microRNA profile in patients with colorectal carcinoma before and after curative surgery. Int J Colorectal Dis.

