# Supplementary material for: MicroRNAs as growth regulators, their function and biomarker status in colorectal cancer
Source: Oncotarget. 2015 Nov 25;7(6):6476–505. doi: 10.18632/oncotarget.6390 (PMC4872728; doi:10.18632/oncotarget.6390)
Supplement: Supplementary file 1 [file oncotarget-07-6476-s001.pdf]

MicroRNAs as growth regulators, their function and biomarker status in colorectal cancer

Supplementary Material

Information retrieval and handling

Studies retrieved from PubMed the 1st of June 2015 combined two search strings, Search 1: "(colorectal neoplasms[MeSH Terms] AND microRNA[MeSH Terms]) NOT review[Publication Type] AND English[Language]" and Search 2: "colorectal cancer" [Title/Abstract] OR colon cancer[Title/Abstract] OR rectum cancer[Title/Abstract] AND miRNA[Title/Abstract], NOT review[Publication Type] AND English[Language]". Output from Searches 1 and 2 were merged and duplicated studies were removed resulting in a total of 1113 studies. All abstracts were read, 178 studies excluded based on manual review, leaving 936 original research articles. The details in the search workflow are illustrated in Supplementary Fig.1 and all included studies are listed in Supplementary Material II. We noted keywords, extracted information about the material (patient samples and cell lines), the reported miRNAs, functionally validated target genes, as well as the pathways affirmed to be regulated by the miRNAs. Keywords were provided in approximately 60% of the studies. To give an overview of all publications, the studies (n=936) were segmented by the most frequently used keywords appearing in a title/abstract in Endnote X6 (Thomson Reuters, Philadelphia, PA). Graphs were prepared in Excel, PASW Statistics 18 (IBM Corp, Armonk, NY) or Bioconductor (version 3.0.2). TCGA CRC patient miRNA and miRNA expression datasets [1] were downloaded at the preprocessed (level 3) from GDAC Broad Institute (analysis run 2014.10.17). Heatmaps constructed including TCGA patients were both miRNAs and mRNAs expression measurements were available using Partek Genomic Suite (6.6). All validated miRNA-mRNA pairs reported in the reviewed studies were used to build a miRNA target network using Cytoscape (version 3.1.0). To avoid redundancy caused by alias, the target gene list was manually curated, annotating all genes with Official Gene Symbol, while miRNA names were kept as they have been reported. The network edges were coded by degree of miRNA-mRNA associations obtained in TCGA CRC patients as reported Li and colleagues[2].

Supplementary references:

1. Cancer Genome Atlas N. Comprehensive molecular characterization of human colon and rectal cancer. Nature. 2012; 487(7407):330-337.
2. Li Y and Zhang Z. Potential microRNA-mediated oncogenic intercellular communication revealed by pan-cancer analysis. Scientific reports. 2014; 4:7097.

3. Chivukula RR, Shi G, Acharya A, Mills EW, Zeitels LR, Anandam JL, Abdelnaby AA, Balch GC, Mansour JC, Yopp AC, Maitra A and Mendell JT. An essential mesenchymal function for miR-143/145 in intestinal epithelial regeneration. Cell. 2014; 157(5):1104-1116.

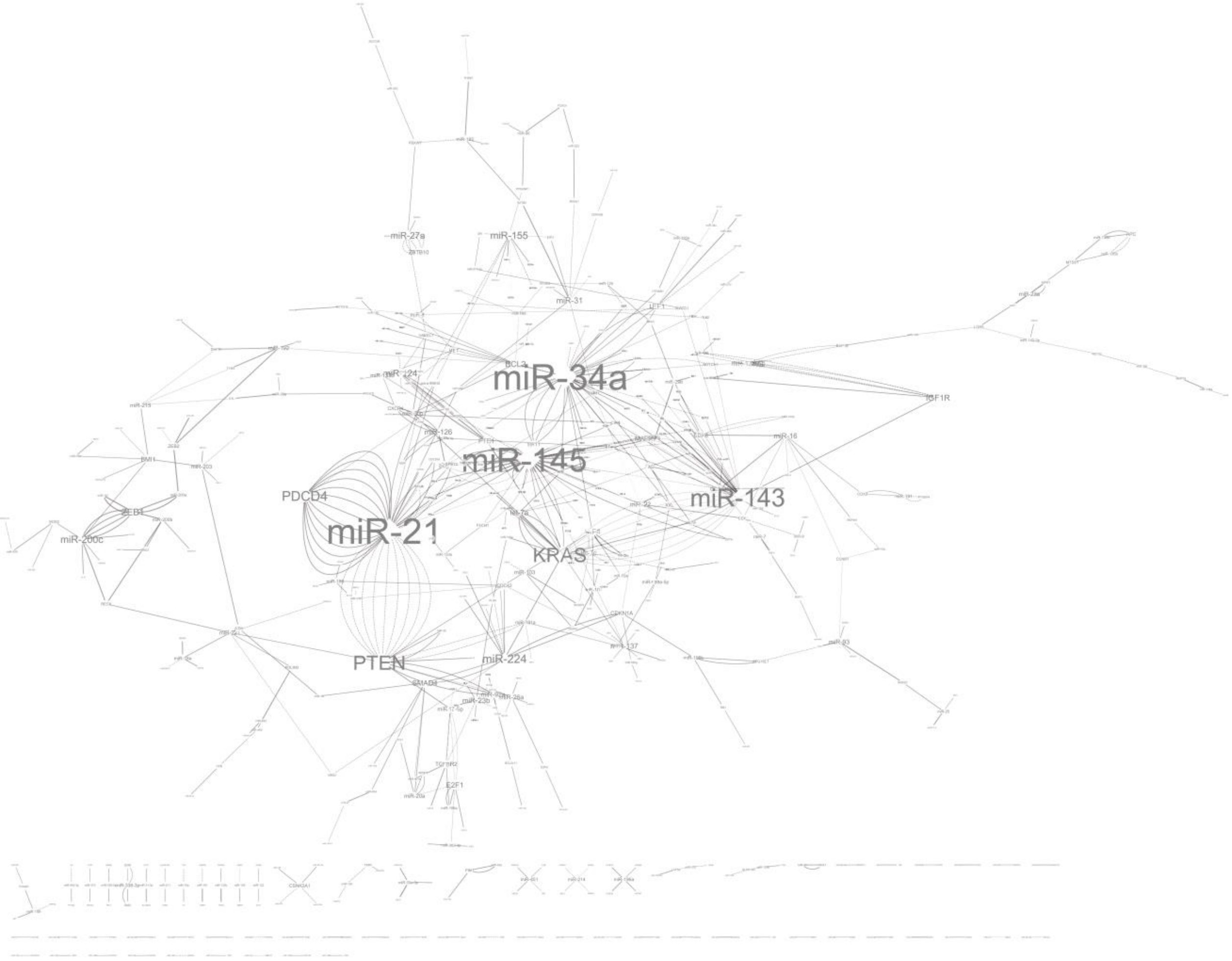

Supplementary Figure1. MiRNA regulation of target genes; extended network.

The miRNA-targets nodes generated using the all validated miRNA-target pairs. The network edges are coded by degree of expressional miRNA-mRNA associations obtained in TCGA CRC patients. MiRNA nodes are labeled as rectangles, target nodes are labeled as ovals. Size of nodes reflects the number of connections, with bigger nodes representing more densely connected regions. The node of miR-143/miR-145-KRAS should be taken with precaution in light of the recent results by Chivukala et. al.[3].
